# Emergent antibiotic persistence in a spatially structured synthetic microbial mutualism

**DOI:** 10.1093/ismejo/wrae075

**Published:** 2024-05-01

**Authors:** Xianyi Xiong, Hans G Othmer, William R Harcombe

**Affiliations:** Department of Ecology, Evolution, and Behavior, BioTechnology Institute, University of Minnesota, St. Paul, MN 55108, United States; Division of Community Health & Epidemiology, University of Minnesota School of Public Health, Minneapolis, MN 55454, United States; School of Mathematics, University of Minnesota, Minneapolis, MN 55455, United States; Department of Ecology, Evolution, and Behavior, BioTechnology Institute, University of Minnesota, St. Paul, MN 55108, United States

**Keywords:** antibiotic persistence, cross-feeding, spatial structure, fluorescent microscopy

## Abstract

Antibiotic persistence (heterotolerance) allows a subpopulation of bacteria to survive antibiotic-induced killing and contributes to the evolution of antibiotic resistance. Although bacteria typically live in microbial communities with complex ecological interactions, little is known about how microbial ecology affects antibiotic persistence. Here, we demonstrated within a synthetic two-species microbial mutualism of *Escherichia coli* and *Salmonella enterica* that the combination of cross-feeding and community spatial structure can emergently cause high antibiotic persistence in bacteria by increasing the cell-to-cell heterogeneity. Tracking ampicillin-induced death for bacteria on agar surfaces, we found that *E. coli* forms up to 55 times more antibiotic persisters in the cross-feeding coculture than in monoculture. This high persistence could not be explained solely by the presence of *S. enterica*, the presence of cross-feeding, average nutrient starvation, or spontaneous resistant mutations. Time-series fluorescent microscopy revealed increased cell-to-cell variation in *E. coli* lag time in the mutualistic co-culture. Furthermore, we discovered that an *E. coli* cell can survive antibiotic killing if the nearby *S. enterica* cells on which it relies die first. In conclusion, we showed that the high antibiotic persistence phenotype can be an emergent phenomenon caused by a combination of cross-feeding and spatial structure. Our work highlights the importance of considering spatially structured interactions during antibiotic treatment and understanding microbial community resilience more broadly.

## Introduction

**Figure 1:**
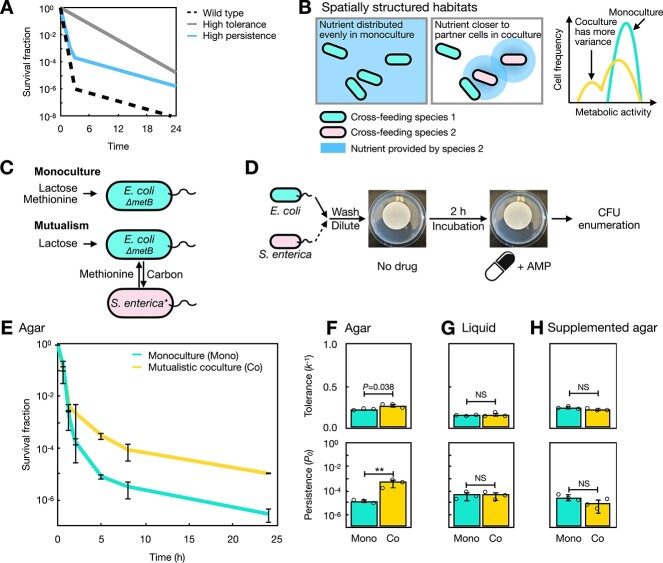
High antibiotic persistence is an emergent phenotype caused by both spatial structure and cross-feeding; (A) survival curves of high tolerance (grey, top line) and high persistence (blue, middle line) bacterial populations under antibiotic killing; **(**B) hypothesis that cross-feeding bacteria in spatially structured habitats have higher variance in metabolic activity due to differential access to nutrient; (C) model system setup; an *E. coli* methionine auxotroph can obtain methionine from media (monoculture) or from a methionine-overproducing *S. enterica* strain (mutualism); the coculture represents an obligate mutualism as *S. enterica* also relies on *E. coli* for carbon in lactose minimal media; (D) monocultures or cocultures were immobilized on nitrocellulose filter membranes and incubated on Hypho minimal agar for 2 h without antibiotics before transferred to agar containing ampicillin (AMP); membranes were disruptively sampled through time to determine survival fractions; (E) *E. coli* survival curves in monoculture and the mutualistic coculture under ampicillin treatment over 24 h; (F) antibiotic tolerance and persistence of *E. coli* in monoculture (mono) and the mutualistic coculture (Co) calculated from (E); (G) *E. coli* tolerance and persistence in shaken liquid in monoculture (mono) and the mutualistic coculture (Co); (H) *E. coli* tolerance and persistence in monoculture (mono) and the methionine-supplemented coculture (Co) on agar; individual measurements from three biologically independent trials (*n* = 3) and their average are shown here and throughout the paper, unless otherwise noted; error bars denote ±1 SD; NS means no significance (*P* > .10); “^*^^*^” means 0.001 *< P* < .01; the same annotation is used throughout the paper.

Understanding strategies of bacteria to overcome antibiotic stress is a pressing task in the 21st century. Antibiotic tolerance and persistence (heterotolerance) are two understudied yet important strategies for bacteria to survive antibiotics [1]. Bacterial populations typically have biphasic survival curves with an initial death rate and a slower secondary death rate (Fig. 1A) [2]. The initial rate of decline is defined by the average death rate for cells in the population, and its inverse can be defined as the tolerance of a population. The secondary rate represents death rate of a subpopulation of cells that can be defined as persisters (Fig. 1A) [2]. Populations can increase survival by altering tolerance, persistence, or both [1–3]. Both tolerance and persistence can contribute to the evolution of antibiotic resistance [4–6], a global public health problem that was associated with ~5 million deaths world-wide in 2019 alone [7]. There is an increasing understanding of the mechanisms of antibiotic tolerance [1, 8, 9], but we remain much more ignorant about the mechanisms behind antibiotic persistence [5, 10].

Antibiotic persistence is challenging to study because persisters are genetically identical to normal cells and typically exist at a frequency <1%. Antibiotic persistence results from phenotypic heterogeneity among cells in a population [11–13], and persistence can arise due to differences in protein activity or gene expression compared to the average cell in the population [14, 15]. These phenotypic differences cause a fraction of bacterial cells to enter a physiological state that allows them to persist through antibiotic killing and recover after the antibiotic perturbation [5, 10, 14–16]. However, we are only beginning to understand ways that growth of bacteria in structured communities with complex ecological interactions [17, 18] impacts antibiotic survival. How microbial ecology contributes to antibiotic persistence remains largely unknown.

Microbial ecology may be important to antibiotic persistence because ecological interactions among bacterial species can affect responses to antibiotic treatment [19–29]. One common ecological interaction is cross-feeding [26, 30–34], which involves bacteria obtaining nutrients from the excretion of other species [35]. Recently, our group showed that cross-feeding can reduce the antibiotic resistance of species down to that of the most susceptible species on which they rely—a mechanism called the “weakest link” hypothesis [19]. Additionally, interactions can alter responses to antibiotics through cross-protection by antibiotic degrading strains [36], induction of efflux activity [26], or altering the pH of growth media [37]. However, there has been little study of the impact of interspecies interactions on antibiotic persistence.

Spatial structure is another ecological factor that can further affect antibiotic persistence in interacting bacteria. One way that spatial structure can generate variation in death rates is by generating differences in the amount of antibiotic each cell experiences. For example, cells deep in biofilms can experience lower concentrations of some drugs and therefore have a higher chance of survival [38, 39]. A second way that spatial structure may generate variation in death rates is by generating differences in physiology between cells [40]. Cells at the center of biofilms often have less access to nutrients, and this reduced metabolic activity can make them less susceptible to antibiotics [38, 41]. Recent research [42] also showed that in an isogenic *Escherichia coli* population within a microfluidic chamber, cells farther away from the nutrient source grow more slowly and die more slowly compared to the rest of the population.

Here, we investigated how the combination of spatial structure and cross-feeding can emergently contribute to antibiotic persistence in bacteria. We hypothesized that growth in a spatially structured environment would lead cells to have differential proximity to cross-feeding partners and thereby differential access to nutrients, driving differential death rates in the presence of antibiotics (Fig. 1B). We used a synthetic obligate cross-feeding mutualism between *E. coli* and *Salmonella enterica* where methionine and carbon are exchanged [43–45], and found that when grown on a surface and exposed to ampicillin, the persister frequency of both species was significantly higher in the cross-feeding coculture than in their respective monocultures. We focused on *E. coli* to understand the mechanism behind this heightened persistence and developed time-series fluorescent microscopy experiments to study growth and death in spatially structured environments on a single-cell level. We discovered that *E. coli* that rely on *S. enterica* can survive if the *S. enterica* cells nearby die first—a novel ecological mechanism underlying persistence in the spatially structured mutualism.

## Materials and methods

### Bacterial strains and culture media

The *E. coli* strain used is a Keio line derivative that carries a *ΔmetB* mutation (JW3910) [46], a reinserted *lac* operon [43], and a genome-integrated, constitutively expressed cyan fluorescent protein (CFP) gene [44]. For data shown in Fig. S3D–G, we used an *E. coli* with a *ΔgalK* mutation and no CFP gene, which is otherwise identical to our *ΔmetB* strain [45] (referred to as “*E. coli ΔgalK*”). The *S. enterica* LT2 strain “*S. enterica*” was a derivative of an experimentally evolved [43] strain that excretes methionine due to a base-pair change in *metA* [47] and an insertion sequence element inserted in front of *metJ* [48]. Here the *S. enterica* strain we used was further engineered to constitutively express a genome-integrated yellow fluorescent protein (YFP) gene [44].

*E. coli* forms an obligate cross-feeding mutualism with *S. enterica* in lactose minimal media [43–45] (Fig. S1A), where *E. coli* cross-feeds methionine from *S. enterica* and secretes a carbon that *S. enterica* requires to grow (Fig. 1C). *E. coli* can also grow in monoculture in the lactose minimal media with methionine supplementation, thus maintaining similar physiology as in the cross-feeding treatment [19] (Fig. 1C and Fig. S1B). The *S. enterica* strain can also be grown and studied in monoculture when supplemented with a carbon source like acetate or galactose (Fig. S1B) [43–45]. In a mutualism between *E. coli ΔgalK* and *S. enterica* in lactose minimal media, *S. enterica* consumes galactose secreted from its partner (Fig. S3C) [45].

Growth and antibiotic killing experiments involving *E. coli* and *S. enterica* took place in Hypho minimal medium. Hypho minimal media is a buffered defined media with 7.26 mM K_2_HPO_4_, 9.38 mM NaH_2_PO_4_, 1.89 mM (NH_4_)_2_SO_4_, 0.41 mM MgSO_4_, 0.6 μM ZnSO_4_, 9.98 μM CaCl_2_, 0.5 μM MnCl_2_, 1 μM (NH_4_)_6_Mo_7_, 0.5 μM CuSO_4_, 1 μM CoCl_2_, 0.169 μM Na_2_WO_4_, 8.88 μM FeSO_2_ [49]. The mutualistic medium or agar (1%) also contains 2.78 mM lactose, whereas the *E. coli* monoculture or methionine-supplemented coculture medium contains 2.78 mM lactose and 0.08 mM methionine. The *S. enterica* monoculture medium contains 16.9 mM acetate or 5.56 mM galactose. Ampicillin sodium salt (Fisher Scientific, MA) was prepared in the agar or added into the liquid media to reach a final concentration of 100 $\mathrm{\mu}$g/ml [50], which is >128-fold higher than the MIC of *E. coli* [19].

The methionine concentration in the *E. coli* monoculture was chosen following previous work as the lowest concentration possible to ensure lactose is the limiting nutrient as in the mutualistic coculture [51, 52]. At the chosen lactose and methionine concentrations, monoculture *E. coli* and the two-species mutualistic coculture reached nearly identical total yields in liquid medium (Fig. S1C).

Fresh (<1 week) *E. coli* and *S. enterica* colonies were inoculated into 5 ml liquid Hypho minimal media with lactose and methionine, and with acetate, respectively. Culture was incubated in 50 ml Erlenmeyer flasks to ensure aeration with shaking at 200 rpm at 37°C till log phase (~14 h for *E. coli* and 24–48 h for *S. enterica*). *E. coli* and *S. enterica* cells were then washed and diluted in saline [53] to OD_600_ = 0.005 or OD_600_ = 0.0025, respectively. For coculture assays, cells were mixed at these OD_600_ to achieve a 1:1 species ratio. The population size for a single species was held constant between monoculture and coculture experiments, meaning that there were twice as many total cells on coculture membranes. We also repeated experiments holding total cell number constant (Fig. S4D and Supplementary Discussion 1).

### Studying bacteria on spatially structured membranes

An amount of 2 ml of the above diluted culture (~5$\times$10^6^ cells/species/membrane) was spread evenly and immobilized on a 0.2 $\mathrm{\mu}$m nitrocellulose filter membrane (4.7 cm diameter; Thermo Fisher Scientific, MA) on a flat-bottom, ethanol sterilized funnel by removing liquid with an air vacuum. In antibiotic killing assays, the membrane was incubated on Hypho agar without antibiotics to allow growth to onset for 2 h, and then a tweezer was used to lift up the edge of the membrane and move it to a different agar plate with 100 μg/ml ampicillin (Fig. 1D and Fig S1D). We expect this movement to have minimal effects on the spatial distribution of microbes. In growth curve assays, the membranes were incubated on Hypho agar without antibiotics.

To determine population sizes at each time point, we disruptively sampled a membrane per biological replicate. Membranes were vortexed for 30 s in 5 ml saline to wash off the cells. For ampicillin killing experiments, a stock solution of 125 unit/ml of β-lactamase (Neta Scientific, NJ) was added to ensure no residual antibiotic [50]. Dilutions were plated on differential Lysogeny Broth (LB) agar with 20 $\mathrm{\mu}$g/ml 5-bromo-4-chloro-3-indolyl-β-D-galactopyranoside (X-gal). After 1 day incubation at 37°C and 5 days at room temperature (~ 24°C), we calculated the population size of *E. coli* and *S. enterica* by enumerating the colony-forming units (CFUs) of blue and white colonies, respectively. The long incubation was to ensure that all colonies—including the small colony variants—appear and form visible colonies with proper color [54].

### Growth physiology measurement

To measure growth of *E. coli* on membrane surfaces, *E. coli* and *S. enterica* cells were distributed on nitrocellulose membranes as described above and incubated until various time points within 48 h. As in the antibiotic killing assays, one membrane per biological replicate was disruptively sampled and vortexed in saline at each time point.

To measure growth of *E. coli* in liquid culture, *E. coli* and *S. enterica* were grown to log phase in their respective minimal media and diluted to OD_600_ = 0.001 in 200 $\mathrm{\mu}$l Hypho minimal media within 96-well plates. Growth at 37°C was measured on a Tecan InfinitePro 200 plate reader (Tecan US, Inc., NC) as OD_600_ for all cells, as CFP signals specifically for *E. coli* (Ex: 430 nm, Em: 480 nm), and YFP signals specifically for *S. enterica* (Ex: 500 nm; Em: 530 nm) every 20 min. This growth experiment was repeated in three biological replicates during three separate weeks.

To calculate growth rate and lag time in liquid or on agar, we fit a custom-built log-linear curve to growth curves composed of CFU/ml, OD_600_, or CFP data. An exponential line was fit to the log phase of the growth curve. The slope of the exponential line was considered growth rate, whereas the time point at which growth starts was considered lag time.

### Antibiotic tolerance and persistence measurement

Survival curves were plotted using the survival fraction data calculated from CFU counts at each ampicillin-treated time point. We fit a biphasic exponential equation to the survival curve for each biological replicate [3, 50]: log_10_(*y*) = log_10_((1-*P_0_*)*e^-kt^* + *P_0_e^-pt^*), where *y* is the survival fraction at each time point (*t*), *P_0_* is the persister fraction, and *k* and *p* are death rates (unit: h^−1^) of normal and persister cells, respectively (Fig. S2, Supplementary Discussion 2). Antibiotic tolerance was measured as *k*^−1^ and persistence as *P_0_* from the biphasic line of best fit (Fig. S2A).

### *E. coli* antibiotic resistance measurement

The minimum inhibitory concentration (MIC) was tested for *E. coli* survivors of antibiotic killing in monoculture and in mutualism with *S. enterica* (*n* = 6 each). After 5-h ampicillin killing, each membrane was moved to a different agar spread with 75 $\mathrm{\mu}$l of 125 unit/ml β-lactamase and incubated for 48 h to obtain full growth, and then washed in 5 ml saline. Each culture was diluted in Hypho minimal media with lactose and methionine to OD_600_ = 0.001 [55] per species against an ampicillin gradient (100 ~ 0.098 $\mathrm{\mu}$g/ml) in duplicates on a 96-well plate, which was incubated with 385 rpm shaking at 37°C for 48 h. Prior work in our group showed that MIC of *E. coli* is twice of *S. enterica* in monocultures [18]. Methionine is supplemented for coculture tests so *S. enterica* MIC should not affect our reading due to its dependency on *E. coli* but not vice versa. *E. coli* MIC was determined for each row as the lowest concentration without growth.

### Time-series fluorescent microscopy

For microscopy, cells were placed on agarose pads (Fig S12). Log-phase *E. coli* and *S. enterica* cultures were washed and diluted to OD_600_ = 0.05 or OD_600_ = 0.025, respectively. This OD_600_ was chosen because prior experiments showed that a volume of 1.5 μl of culture at this dilution level onto a dry Hypho minimal agarose pad (W: 0.4 cm $\times$ L: 0.4 cm $\times$ H: 0.1 cm) with 1% (m/v) agarose (Sigma Aldrich, MO) leads to similar total density under the microscope as in the agar experiment. The droplet was dried at room temperature on the agarose pad for 10 min, which was then flipped over onto a microscope slide (ibidi USA Inc., WI) so bacteria were between the pad and the slide [56]. The pads were then incubated for 2 h at 37°C in the microscope chamber on the Nikon A1si Confocal and Widefield inverted microscope in the University Imaging Centers at the University of Minnesota (St. Paul, MN). For antibiotic experiments, a drop of 1.5 μl ampicillin solution was added on top of the agarose pad on the microscope before imaging such that the pad obtained a drug concentration of 100 μg/ml.

We programmed the Nikon Elements v5.41 software to collect fluorescent signals with 200× total magnification (10 × eye piece and 20× objective magnifications on an inverted microscope) at the center of each agarose pad. The images were taken with perfect focus (PFS) every 20 min for a total of 7 h in two fluorescent channels: 488 nm (CFP) for *E. coli* and 514 nm (YFP) for *S. enterica*. When taking large microscopic images in the antibiotic killing experiments, we used the Large Images option in the Nikon Elements software to automatically stitch nine 160 μm × 160 μm images together at each time point. Stitching was done with 1% edge distance overlap between two adjacent images, resulting in a total image of 476 μm × 476 μm. All images were taken with PFS on, which, according to the manufacturer manual, should not result in visible change of magnification over time.

The collected time-series images were then aligned and noise was removed using the software. In particular, a rolling-ball algorithm (radius: 1.27 μm) was implemented around each bright object to subtract background. An automatic deconvolution method was used to enhance contrast and remove blur (modality: Point scan confocal; pinhole size: 93.23 μm; magnification: 20.0$\times$; numerical aperture: 0.75; immersion reference index: 1.0 [air]; calibration: 0.156 μm/pixel). Local contrast was set at 25% for a radius of 2.34 μm per object. Single cells were identified by detecting regional maxima at the center from a 5$\times$5 matrix. The fluorescent signal threshold was set to 100 unit of intensity to produce binary images of individual cells, which were objects that passed the threshold. Cells on the image borders were also removed from analysis. Images were then further analyzed using ImageJ (FIJI, v1.53k) [57].

For the growth analysis, images were in a dimension of 160 μm $\times$ 160 μm and we initially added ~100 cells per species per image in the beginning of the experiment. ImageJ was used to identify cell clusters as nearby cells that image processing above could not separate. We then took the images from the first 3 h for analysis because clusters started to fuse together after then due to growth, which made growth rate calculations difficult. The cell cluster biomass was defined as the fluorescent signal area per cluster. We identified the minimal time frame at which each cluster gains a biomass of 10%, which we considered lag time in a similar process following previous work [4, 8, 40, 54].

For the death analysis, images were usually in a dimension of 476 μm × 476 μm with 500–900 cells per species per image in the beginning of the experiment. We used ImageJ to measure single object area (biomass) at each location over time. Objects with >25 pixels of their respective fluorescent signals were considered cells. We considered the single-cell death time as the earliest time frame for a cell to disappear from the microscopic view.

### Statistics

One-way analysis of variance (ANOVA) tests, linear regression, *F*-tests, and Spearman’s correlation tests were implemented in R v4.3.3 [58]. Unless noted, the reported *P* values are from one-way ANOVA tests run on data from three biological replicates (*n* = 3). For multiple comparisons tested with one-way ANOVA, we reported *P* values adjusted with Tukey’s honestly significant difference (HSD) [59]. The minpack.lm package (v.1.2-4) was used to perform the biphasic exponential fit to survival curves.

## Results

### Spatially structured cross-feeding causes heightened antibiotic persistence in our system

To investigate whether cross-feeding affects bacterial response to antibiotic killing on spatially structured surfaces, we incubated *E. coli* in monoculture and in the cross-feeding coculture with *S. enterica* on agar plates and measured its survival curves when treated with high-dosage ampicillin (Fig. 1C and D and Fig S1D). We found that *E. coli* has different, biphasic survival curves in monoculture and in mutualism (Fig. 1E). Although *E. coli* has a small difference in tolerance between the monoculture and mutualistic conditions (*P* = .038), the mutualistic *E. coli* has up to 55-fold more antibiotic persisters in coculture (*P* = .0025; Fig. 1F). The increased persistence in mutualism was also generally true for *S. enterica* (Fig. S3). Thus, cross-feeding on spatially structured surfaces can increase antibiotic persistence in our system.

To understand our observation of higher *E. coli* persisters in coculture, we focused on *E. coli* and tested whether cross-feeding is sufficient to drive the high antibiotic persistence. We repeated the experiment in shaken liquid without spatial structure [42, 43] and maintained similar average cell-to-cell distances as on surfaces (Fig. S4A and B; Supplementary Discussion 1). We found that in liquid cultures with ampicillin, *E. coli* has similar antibiotic tolerance (*P* = .60) and persistence (*P* = .97) in monoculture and the mutualistic coculture (Fig. 1G). We also observed that the monoculture persister fractions do not differ greatly between agar and liquid environments (Fig. S4C), suggesting that moving from liquid culture to agar alone does not drive high persistence.

We examined whether cross-feeding is essential for the high *E. coli* persistence. When the metabolic dependency of *E. coli* on *S. enterica* was broken by supplementing methionine in the agar, we observed that *E. coli* does not show the high persistence phenotype without cross-feeding with *S. enterica* for methionine (*P* = .15; Fig. 1H). Together, all data above suggest that cross-feeding and spatial structure are both essential contributing factors to high antibiotic persistence in *E. coli*.

### High *E. coli* antibiotic persistence on mutualistic agar is not due to slower growth rate, strong methionine starvation, or antibiotic resistance

**Figure 2:**
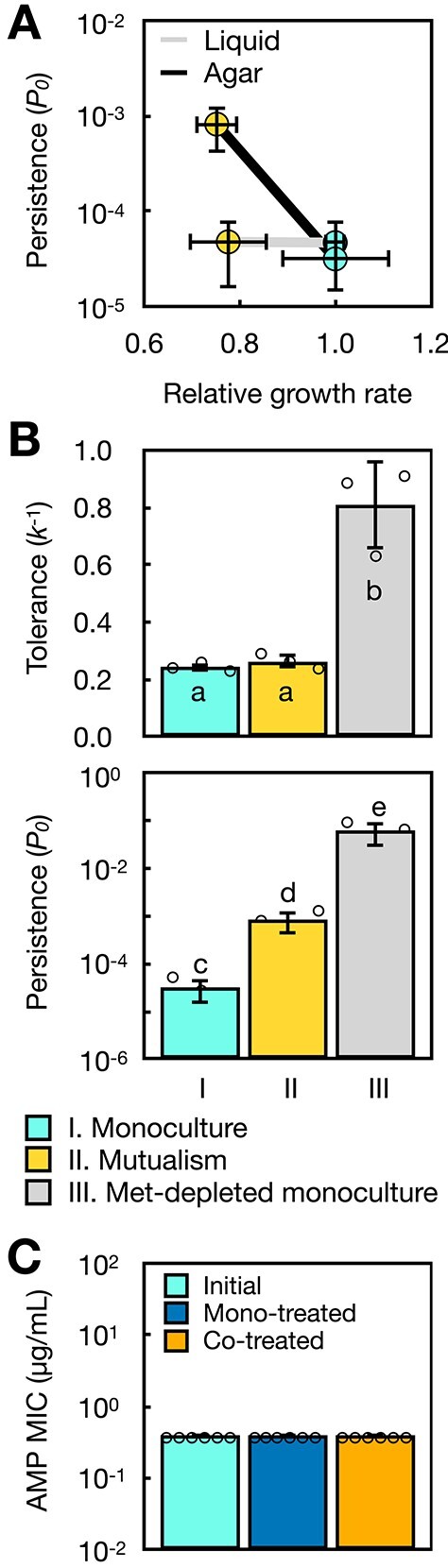
High persistence for *E. coli* cross-feeding on a surface is not driven by slower growth, average methionine starvation, and antibiotic resistance; (A) *E. coli* on average grows 25% more slowly when cross-feeding than in monoculture in liquid (grey line) and on agar (black line), but higher *E. coli* persistence is only visible on mutualistic agar; (B) tolerance and persistence for monoculture *E. coli* tested on methionine (met) depleted agar (III); lower case letters indicate treatments with no detectable statistical difference with Tukey’s HSD *P* > .1; this annotation is used for the rest of the paper; (C) MIC measurements for *E. coli* survivor populations (*n* = 6) after 5-h ampicillin (AMP) treatment in monoculture (mono-treated) and mutualism (Co-treated) agar following Fig. S6; initial: untreated *E. coli* population controls.

We investigated whether growth rate was the primary driver of increased persistence when *E. coli* cross-feeds on a surface. Slower growth has been associated with increased survival [8], and our *E. coli* grows 25% slower when cross-feeding than in monoculture on a surface (Fig. 2A and Fig. S5). However, in liquid, *E. coli* grows similarly slower when cross-feeding than in monoculture, yet we did not observe difference in persistence there (Figs 1G and 2A). Consistent with these observations, *E. coli* growing at a lower rate on monoculture agar at a low temperature does not have significantly increased persistence (Fig. S5B).

We examined whether starvation [1, 9, 11, 14, 53] of the methionine nutrient causes more *E. coli* persistence when cross-feeding on a surface. When *E. coli* monocultures were tested on agar without methionine, we observed a significant increase in persistence (pairwise Tukey’s HSD *P* < .00022). However, we also observed a significant increase in tolerance under methionine starvation (pairwise Tukey HSD *P* < .00080, Fig. 2B). We also found that increasing methionine starvation in liquid by lowering density of cross-feeding coculture to 1% led to little increase in *E. coli* survival against ampicillin killing (Fig. S4E). These data suggest that average starvation for methionine can explain increased tolerance but not persistence when *E. coli* is cross-feeding on a surface.

To confirm that the *E. coli* survivors of antibiotic treatment in structured coculture were persistent rather than resistant, we measured antibiotic resistance of *E. coli* populations regrown from survivors after 5-h ampicillin treatment in monoculture and mutualistic coculture (Fig. S6). We found no evidence of increased ampicillin resistance in *E. coli* in either condition (Fig. 2C).

### Cross-feeding in structured habitats increases lag time heterogeneity in *E. coli*

**Figure 3:**
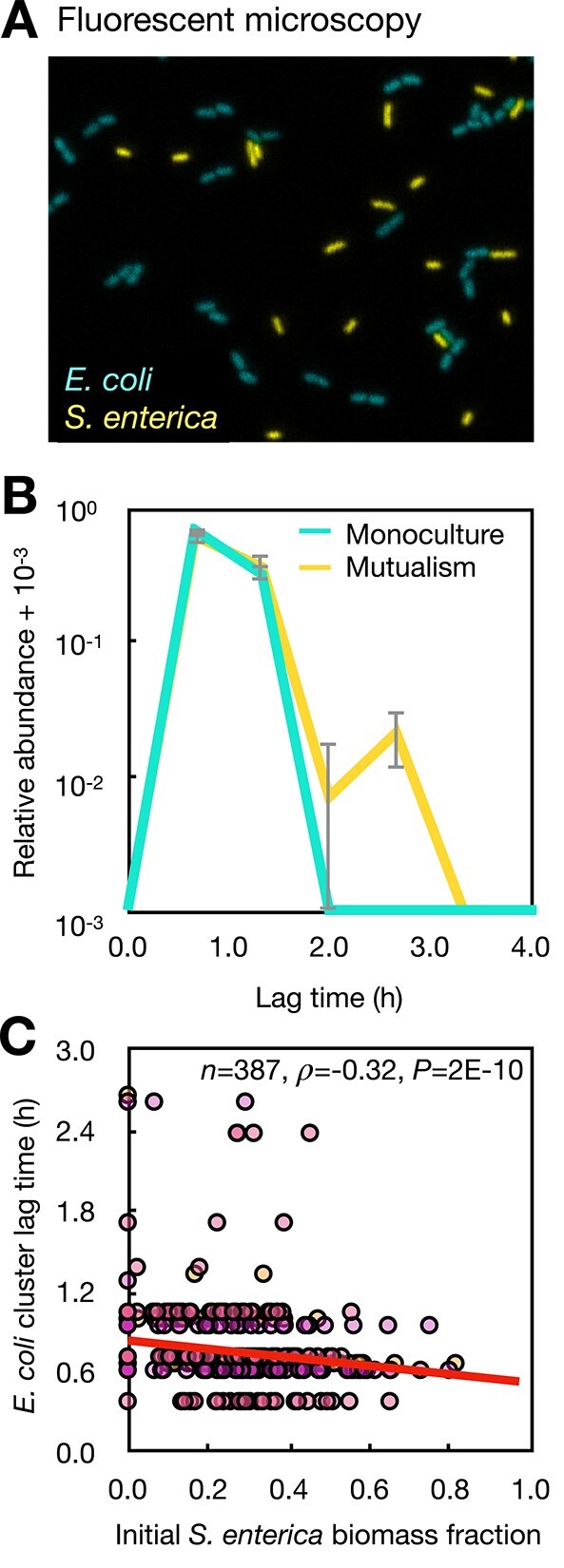
Cross-feeding and spatial structure increase cell–cell heterogeneity in the *E. coli* lag time during growth; (A) a time-series fluorescent microscopy experiment was done to track growth for each single *E. coli* cell cluster; (B) distributions of the *E. coli* lag times measured in monoculture and in the mutualistic coculture with *S. enterica* on Hypho minimal agarose pads; *Y*-axis is log-transformed; (C) the initial biomass fraction of *S. enterica* within a small (radius = 8.6 μm) neighborhood of each *E. coli* cell cluster can predict lag time of *E. coli* clusters (Spearman’s correlation on top of figure); data from all cells on three agarose pads (*n* = 3) are included. A linear regression model fit is also shown (slope = −0.44, adjusted *R*^2^ = 0.048, *P* = 7.7E-6).

We hypothesized that cross-feeding and spatial structure together increase the cell-to-cell heterogeneity in the *E. coli* growth. To test this hypothesis, we used time-series fluorescent microscopy to directly observe *E. coli* on a single-cell level as it grows on spatially structured agarose pads [56], and measured lag time for single cell clusters (Fig. 3A and Fig. S7A). The majority of *E. coli* clusters exits lag phase within <2 h (Fig. 3B). In cross-feeding cocultures, about 1% of *E. coli* stays in lag for 2–3 h (*y*-axis is log-transformed), significantly increasing variance in the lag time distribution in the mutualistic coculture among all cell clusters studied across three independent agarose pads (*F*-test, *P* < 1.4E-14; Fig. S7B). This result is consistent with the increased persistence in the population-level measurement on agar (Fig. S5), and is also supported by a mathematical model on individual colony growth (Fig. S8, Supplementary Method 1 and 2, Supplementary Discussion 3, and Table S1). Together, these data encouraged us to ask why there is higher lag time variation in the mutualistic coculture.

We proposed that the spatial structure of the cross-feeding mutualism determines the *E. coli* lag times. To confirm the insight from previous work that cross-feeding bacteria on a surface interact in local neighborhoods within a short range [43–45, 60], we experimentally tested whether each *E. coli*’s lag time can be predicted by its proximity to the cross-feeding *S. enterica* partners. We validated that in a neighborhood with a radius of ~10 $\mathrm{\mu}$m around each *E. coli* cluster, higher fractions of the *S. enterica* biomass in the beginning of the experiment correlate with shorter *E. coli* lag times (Spearman’s $\rho$= − 0.32, *P* = 2E-10; Fig. 3C). Furthermore, the above correlation becomes weaker as we considered larger neighborhood sizes (Fig. S9). Together, we conclude that the initial spatial distribution of cells in the cross-feeding mutualism can explain the increased lag time heterogeneity, which may be key to having more persisters.

### Fluorescent microscopy revealed an unexpected mechanism behind high *E. coli* persistence in the cross-feeding mutualism

**Figure 4:**
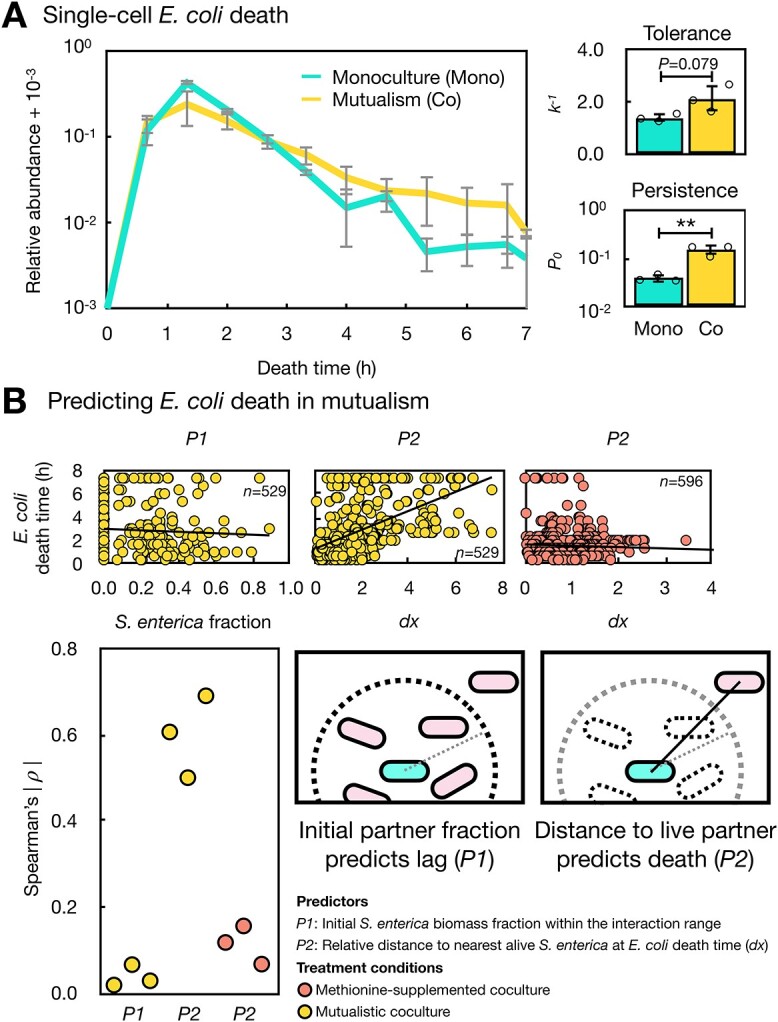
Loss of access to nutrient underlies high *E. coli* persistence in the structured mutualism; (A) death time distributions of *E. coli* during ampicillin treatment within 7 h of imaging across three independent agarose pads (*n* = 3, left); tolerance and persistence measurements for *E. coli* on each pad following Fig. S2A using survival curves in Fig. S10B (right); (B) the absolute Spearman’s $\rho$ in each agarose pad was plotted for predictors (*P1*, *P2*) of the single-cell *E. coli* death time in the mutualistic or the methionine-supplemented coculture in the bottom left; representative raw correlation plots using either predictor for one biological replicate are shown on the top with total cell number listed (*n*), and linear regression model fits. Schematic: interaction range around a representative *E. coli* cell (center of circle) is shown as a dashed circle; all other cells in each plot are *S. enterica*; dashed line: radius of interaction range; solid line: distance from *E. coli* to live *S. enterica.*

We established an antibiotic killing protocol on spatially structured agarose pads with time-series fluorescent imaging (Fig. S10A). Ampicillin-induced death in *E. coli* measured by fluorescence on the microscope matched that measured by CFU count (Fig. 4A and Fig. S10B–E). We found that the individual *E. coli* death time did not correlate with the initial fraction of *S. enterica* biomass around each *E. coli* cell (Spearman’s $\rho$=0.00661 ± 0.0509, *n* = 3; predictor *P1*, Fig. 4B) or the initial distance to the nearest *S. enterica* neighbor (Spearman’s $\rho$=0.000701 ± 0.0400, *n* = 3). Thus, although the initial spatial structure of the mutualism is sufficient to predict lag time (Fig. 3C), it is insufficient to explain the higher persister fraction.

We tested whether the dynamic change of spatial structure in mutualism over time determines the *E. coli* single-cell death time. Because *S. enterica* are killed by ampicillin as well, perhaps it is crucial to consider proximity over time rather than just the initial proximity of *E. coli* to its partner. At the time of death for each *E. coli* cell, we determined the distance to the nearest alive *S. enterica*. We then standardized this distance against the average distance between partners within the microscopic frame at each time point (Supplementary Discussion 1), in order to prevent artificial correlations when fewer cells are present in the microscopic frame at later time points. We found that taking *S. enterica* death into account in this way leads to a much stronger correlation between proximity and death time (Spearman’s $\rho$=0.598 ± 0.0953, *n* = 3; predictor *P2*, Fig. 4B). Thus, this result confirms a strong spatial pattern contributing to the increased *E. coli* persistence in the mutualism.

We tested the predictability when *E. coli* is challenged with ampicillin in coculture but not cross-feeding with *S. enterica*. We found that in the methionine-supplemented facilitative coculture, the *E. coli* death time barely correlates with the standardized distance to the alive *S. enterica* neighbor (Spearman’s $\rho$=0.118 ± 0.0519, *n* = 3, predictor *P2*, Fig. 4B). When we removed the standardization procedure in generating *P2*, the predictability increases greatly for *E. coli* (Fig. S11A), but all predictors work less well for *S. enterica* (Fig. S11B). Together, we show that the temporal dynamics of the cross-feeding community spatial structure determines the *E. coli* death time throughout ampicillin treatment. *E. coli* cells lose access to methionine as their *S. enterica* neighbors die and this loss increases *E. coli*’s chance of survival in the ampicillin treatment (Fig. 4B).

## Discussion

In this work, we demonstrated that cross-feeding in a spatially structured environment can increase antibiotic persistence in both the mutualistic *E. coli* and *S. enterica* as compared to their respective monocultures. We found that the high persistence phenotype in *E. coli* is an emergent property that appears when cross-feeding takes place in a structured habitat. Effects of cross-feeding on average growth rate, average starvation for methionine, or spontaneous resistant mutations do not appear to be primary drivers of this effect. Observing individual *E. coli* cells under the microscope, we found a higher variation in the lag time in the mutualistic coculture that can be attributed to the initial proximity to mutualistic partners. However, this initial spatial structure cannot predict the single *E. coli* cell death time. By directly observing ampicillin-induced death in *E. coli* and *S. enterica* cells, we discovered that the *E. coli* that relies on *S. enterica* can survive the antibiotic killing if the *S. enterica* cells nearby die first—a novel ecological mechanism contributing to antibiotic persistence.

A key discovery in our work is that cross-feeding in structured habitats can result in more persisters. Antibiotic persistence has been shown to be the result of cell-to-cell variation in physiology in a population [1, 3, 5, 13–16, 53, 54, 61]. This phenotypic variation can arise from stochastic differences in transcription [15, 16] or translation [14] levels among cells. Our observation suggests that cross-feeding on a surface introduces additional cell-to-cell variation as a result of stochastic differences in how close a cell lands to its cross-feeding partners. In liquid cultures nutrients diffuse rapidly, reducing environmental heterogeneity and the stochastic effects of location. High antibiotic persistence is therefore an emergent property resulting from the interplay between two important ecological factors, interspecies interactions and spatial structure (Fig. 1).

Variation in lag time can be predicted by the frequency of partners in a small neighborhood (Fig. 3). Growth on surfaces typically makes interactions local [36, 40, 43–45, 60], and our results suggest that cross-feeding was strongest among cells within ~10 $\mathrm{\mu}$m in our current experiments. This result is in agreement with previous work [60] suggesting that growth of cross-feeding *E. coli* is predicted by the fraction of partners within 3–12 $\mathrm{\mu}$m. These results highlight that the location of bacteria is critical for understanding which cells interact in microbial systems.

The lag time variation caused by initial partner proximity was insufficient to predict the *E. coli* death time (Fig. 4B). Death of cells quickly alters the proximity of cross-feeding partners, so it is critical to account for this loss of access to nutrient (Fig. 4B). We would have missed this critical mechanism if we assumed that the subpopulation behavior in bacterial cells during growth can predict their death patterns as in previous research [4, 8, 10, 16, 42, 53, 60–62]. A few studies [42, 50, 53, 62] did directly observe the death time of individual bacterial cells in antibiotics. Studies [8, 50] that have measured death directly do often find correlation between growth and death, suggesting that the lack of correlation we observed is likely due to our interspecies interactions. Strikingly, our microscopy experiment (Fig. 4) suggests that the cross-feeding *S. enterica* must continually produce methionine to support the growing *E. coli* cells in the neighborhood. This result emphasizes that location is not a static feature of microbial communities but rather a dynamic attribute that changes with birth and death of cells.

The current work indicates that the antibiotic persistence in human-associated microbes may be higher than monitored in clinical laboratories. Lab studies often exclude the spatially structured interspecies interactions that are prevalent in the human microbiome [63]. There is growing appreciation that cross-feeding may be more common in the human body than previously thought (e.g. *Pseudomonas aeruginosa* cross-feeds with the mucin-degrading anaerobes in the Cystic Fibrosis lungs [64]). Although previous work assumed that mutualism or spatial structure is not common in the gut [65, 66], recent evidence showed that metabolite exchange and cross-feeding are common in bacteria in nature and in hosts [34]. The mammalian gut microbiomes are also found to be highly structured [67–69] especially on the mucus layer [70]. Furthermore, consuming human therapeutic drugs can induce cross-feeding in the gut microbiota [71]. Our results thus imply that antibiotic persistence in the human-associated microbiota is underestimated.

Our study has several limitations. Heightened antibiotic persistence was observed in our synthetic, two-species obligate mutualism exchanging methionine and carbon on a surface (Fig. 1 and Fig. S3), and it is unclear the extent to which our findings will extend to other systems. Methionine is the start codon for all proteins, so whether the high persistence also appears in mutualisms cross-feeding other amino acids [52, 72, 73] remains untested. Even within our system, loss of access to nutrient due to partner death works better for predicting *E. coli* death than for *S. enterica* (Fig. 4), presumably because methionine production is dependent on *S. enterica* growth but carbon production can happen in non-growing *E. coli*. Furthermore, our findings are specific to ampicillin, a β-lactam drug just like penicillin that targets growing cells [74]. Whether the heightened persistence phenotype will appear when exposed to other antibiotics [75] is also unknown.

In the current study, we show that microbial ecology can affect antibiotic persistence. Our work highlights that microbial interactions and spatial structure can generate emergent increase in individual heterogeneity. As we work to develop precision management of human microbiomes, it will be critical to continue to advance our understanding of the scale over which interactions occur, and how these interactions shape the behavior of individual cells, populations, and communities.

## Supplementary Material

Xiong_supplement_final_wrae075

## Data Availability

Figures were generated using Numbers v.11.2 (Mac OS). The datasets generated during and/or analyzed during the current study and the custom code are available at https://github.com/xion1475/persistence_ecology. Raw microscopic images are available in the Zenodo repository at doi.org/10.5281/zenodo.10975976.
