# Supplementary material for: Emergent antibiotic persistence in a spatially structured synthetic microbial mutualism
Source: ISME J. 2024 May 1;18(1):wrae075. doi: 10.1093/ismejo/wrae075 (PMC11104777; doi:10.1093/ismejo/wrae075)
Supplement: Xiong_supplement_final_wrae075 [file xiong_supplement_final_wrae075.pdf]

Supporting Materials for

Emergent antibiotic persistence in a spatially structured synthetic microbial mutualism

Xianyi Xiong, Hans G. Othmer, William R. Harcombe

This PDF file includes:

- Supplementary Mathematical Models 1-2
- Supplementary Discussion 1-3
- Supplementary Figures S1-S12
- Supplementary Table 1
- Supplementary References

For source data see attached Excel file "Xiong_Raw_Data.xlsx"

Supplementary Mathematical Models

1. PDE-based biophysical model of bacterial colony growth

Model setup: We developed a mathematical model using partial differential equations (PDEs) to computationally simulate the growth of individual *E. coli* and *S. enterica* colonies in monoculture and the mutualistic coculture on a 1-dimension line or on a 2-dimension grid.

In the resource-explicit model, bacterial colonies interact with local nutrients in a spatially structured environment to grow. On a 1-dimension line of distance $Y = 5$, individual cells of *E. coli* (E) and/or *S. enterica* (S) were seeded randomly by setting the initial conditions at $t = 0$ to be non-zero at random locations, x, whereas various nutrients were initially overlaid evenly on the entire line. This random seeding of bacterial cells makes the PDE model able to describe bacterial populations and communities with spatial structure. Each PDE in Equations [1~2] denote the rules regarding how abundance of bacteria or nutrients changes over 40 units of time, $t \in [0,40]$, at any particular x.

Equations in [S1] denote how *E. coli* and *S. enterica* cross-feed and grow in a mutualistic community. First, the growth of *E. coli* and *S. enterica* follow Eqs. [S1a] and [S1b], respectively. *E. coli* grows by consuming methionine (M) and lactose (L), whereas *S. enterica* grows by taking in acetate (A). The maximum growth rates are r_E and r_S, respectively, and the instantaneous growth rates are limited by the nutrient's local concentration in a Monod fashion with the half-saturation constants, K_M, K_L, and K_A. Growth is ultimately limited by exhaustion of nutrient explained below. *E. coli* growth rates depend on both methionine and lactose, whereas *S. enterica* growth rates depend only on acetate (see Model Assumptions).

$$\frac{\partial E}{\partial t} = r_E\left(\frac{M}{M+K_M}\right)\left(\frac{L}{L+K_L}\right)E - \kappa_E E. \qquad \text{[S1a]}$$

$$\frac{\partial S}{\partial t} = r_S\left(\frac{A}{A+K_A}\right)S - \kappa_S S. \qquad \text{[S1b]}$$

Eq. [S1c] denotes the temporal dynamics of methionine. As a nutrient source, methionine diffuses in space over time at a rate, D_M. It is produced by *S. enterica* at a rate, p_M, and is proportional to *S. enterica*'s consumption of acetate in a Monod fashion (Hammarlund et al., 2021). Methionine gets consumed by *E. coli* following a Monod kinetics by a maximum rate, c_E. Finally, methionine degrades slowly over time at a rate, κ_M.

$$\frac{\partial M}{\partial t} = D_M\nabla^2 M + p_M r_S\left(\frac{A}{A+K_A}\right)S - c_E\left(\frac{M}{M+K_M}\right)\left(\frac{L}{L+K_L}\right)E - \kappa_M M. \qquad \text{[S1c]}$$

Similar to methionine, acetate diffuses at a rate, D_A, gets consumed by *S. enterica* following Monod kinetics, decays exponentially by κ_A (Eq. [S1d]). Acetate is produced by *E. coli* at a rate, p_A, as both lactose and methionine are being consumed. Acetate concentration decreases due to *S. enterica* consumption by a maximum rate, c_S.

$$\frac{\partial A}{\partial t} = D_A\nabla^2 A + p_A r_E\left(\frac{M}{M+K_M}\right)\left(\frac{L}{L+K_L}\right)E - c_S\left(\frac{A}{A+K_A}\right)S - \kappa_A A. \qquad \text{[S1d]}$$

Finally, the temporal dynamics of lactose concentration—which is only consumed by *E. coli*—follows Eq. [S1e]. Here, the natural decay rate of lactose is, κ_L.

$$\frac{\partial L}{\partial t} = D_L\nabla^2 L - c_L\left(\frac{M}{M+K_M}\right)\left(\frac{L}{L+K_L}\right)E - \kappa_L L. \qquad \text{[S1e]}$$

The Neumann boundary conditions are:

$$\frac{\partial N}{\partial x} = 0 \text{ at } x = 0,\ 5, \qquad \text{[S1f]}$$

for $N \in \{E, S, M, A, L\}$.

To simulate *E. coli* grown in monoculture, we remove *S. enterica*- and acetate-associated terms from Eqs. [1] to form Eqs. [2] below, as methionine and lactose are now supplemented in the growth medium for monoculture *E. coli* and so no production terms are necessary. All variables are identical to those in Eqs. [1].

$$\frac{\partial M}{\partial t} = D_M \nabla^2 M - c_E \left(\frac{M}{M + K_M}\right)\left(\frac{L}{L + K_L}\right) E - \kappa_M M, \qquad \text{[S2a]}$$

$$\frac{\partial E}{\partial t} = r_E \left(\frac{M}{M + K_M}\right)\left(\frac{L}{L + K_L}\right) E - \kappa_E E, \qquad \text{[S2b]}$$

$$\frac{\partial L}{\partial t} = D_L \nabla^2 L - c_L \left(\frac{M}{M + K_M}\right)\left(\frac{L}{L + K_L}\right) E - \kappa_L L. \qquad \text{[S2c]}$$

Similarly, the boundary conditions are:

$$\frac{\partial N}{\partial x} = 0 \text{ at } x = 0,\ 5, \qquad \text{[S1f]}$$

for $N \in \{E, M, L\}$.

<u>*Model assumptions:*</u> Our PDE models above are based on the following assumptions.

1. Bacterial growth is determined by 3 nutrients (methionine, lactose, and acetate). In experiments, more nutrients sources have to be present to support growth. For example, ammonia is present in the medium for both monoculture and mutualistic coculture. But, the impact of these other nutrient was factored into the growth rate variables r_E and r_S.

2. Nutrient production of both *E. coli* and *S. enterica* only occurs when their growth nutrients are present. In reality, *E. coli* initiates the mutualism by producing acetate without having to metabolize on methionine (unpublished data). This assumption allows us to keep the model simple without having to make special mathematical treatment for *E. coli* and is also a convention in modeling the cross-feeding growth (e.g. <u>Hammarlund et al., 2021</u>).

3. Bacterial growth leads to no diffusion of biomass. In reality, bacterial growth on agar surfaces results in colony formation and colony size expansion (Chacón et al., 2018). This can change the average distance among bacterial colonies.

4. The growth rate of each colony reflects the instantaneous growth rate of individual cells on the nitrocellulose membrane before ampicillin treatment in the experiment.

5. Bacteria naturally die and nutrients naturally decay over the course of the modeling. These decays are linear with respect to the instantaneous abundance of the bacteria or the nutrient.

6. The resource-explicit growth of *E. coli* is based on the product of the two Monod terms, and not the minimum of the two as in Hammarlund et al. (2019).

<u>*Model implementation & parameterization:*</u> Twenty locations on the 1D line or in the 2D grid were selected randomly to seed *E. coli* (and *S. enterica*, if in the mutualistic coculture) colonies in the form of biomass at the initial time point, and nutrient was distributed evenly on the line as well. All variables in the equations above take values in Supplementary Table 1, which our research group together curated based on the previous ODE model

describing the *E. coli* and *S. enterica* growth in our cross-feeding system (e.g. Hammarlund et al.; 2019; Hammarlund et al., 2021). The diffusion constants of nutrients were estimated based on another previous work in our group (Chacón et al., 2018). On the 1D line, the initial colony biomass was set at 20 locations to be $E\left(t = 0, x_i\right) = 100$ and $S\left(t = 0, x_j\right) = 100$ for *E. coli* and *S. enterica*, respectively, at locations x_i or x_j. In the 2D grid, colonies were seeded at the same expected frequency for *E. coli* and *S. enterica* as in the 1D model.

Very little (but non-zero) abundance of methionine and acetate were seeded evenly on the 1D line in the PDE model for the cross-feeding coculture. This follows previous convention in modeling the growth of an obligate cross-feeding mutualism (Sun et al., 2019; Harmmarlund et al., 2021). Briefly, the initial lactose was set to be $L\,(t = 0, x) = 1000\,\forall x$, methionine and acetate was seeded at $M\,(t = 0, x) = 0.01$ and $A\,(t = 0, x) = 0.01$ for all x on the entire 1D line. Similarly in the monoculture model, the initial lactose and methionine concentrations were $L\,(t = 0, x) = M\,(t = 0, x) = 1000$ for all x. No acetate or *S. enterica* was present in the monoculture model.

Specifically for the 2D simulations, we used the finite difference method to discretize space in our PDEs, and solved the equations using in-house code in R v.4.3.3 (R Core Team, 2024). All boundary conditions apply to the edge grids.

Then, we non-dimensionalized the system in Eq. [S1] and [S2] as in Supplementary Discussion 2. We numerically solved the non-dimensionalized system Eq. [S3~S4] using MATLAB R2021b with the *pdepe* function for the 1D model. Finally, we calculated the growth rates and lag time of individual colonies by fitting a log-linear growth curve to the log-transformed biomass using in-house code in R.

2. ODE-based antibiotic killing in populations with persisters

<u>*Model setup & implementation:*</u> We let the unitless fractions of the non-persisters and persisters in a population of bacteria be denoted by constants n and p, respectively. We first calculated the MDK99 measurements of the population in ampicillin killing as p changes. To do so, we first measured the persister fraction, p, of the WT bacterial population experimentally (Materials & Methods), and then calculated the death rates of the non-persisters and persisters by taking the slopes of the respective log-transformed death phases with respect to the drug treatment time, t. The death rates were denoted by $\kappa_n < 0$ and $\kappa_p < 0$, respectively. We always keep $\kappa_n < \kappa_p$, given that persisters die more slowly than non-persisters. Afterwards, we simulated the kill curve by ampicillin in a population with persister, p, with a set of simple ODEs:

$$\frac{dN}{dt} = \kappa_n N,$$
$$\frac{dP}{dt} = \kappa_p P, \qquad \text{[S5]}$$

for the dynamics of survived non-persisters (N) and survived persisters (P) over time t.

To simulate antibiotic killing dynamics, we took $\kappa_n = -4.2829$ and $\kappa_p = -0.3233$ by fitting kill curves of the non-persisters and persisters in the experimental antibiotic killing assay for the monoculture *E. coli* on agar (Fig. 1C-D). With various initial time points $N_0 \equiv n$ and $P_0 \equiv p$, we analytically solved the system in Eq. [S5], and plotted $(N + P)$ with respect to t for several total persister fractions p (Fig. S2B). Then, we plotted the relationship between the measured MDK99 and p in a population with susceptible (WT) cells and persisters at different p, shown by the function MDK99 $= f(p)$ in Fig. S2C.

Supplementary Discussion

1. Determining cell-cell distance in shaken liquid and on agar surfaces

Here, we determined the average cell-cell distance on agar surfaces (in liquid) by assuming that cells are uniformly distributed in the 2D (3D) space. Each cell is assumed to be at the center of a very small square (lattice) with equal size. And the average cell-cell distance is estimated to be the size of the square (lattice).

We showed that the average cell-to-cell distances in shaken liquid (5×10^7 CFU/mL) and on the nitrocellulose membrane surfaces (5×10^6 CFU/membrane) are comparable (Fig. S4B):

- Monoculture on Membranes: 5×10^6 *E. coli* CFU/membrane (OD_{600}=0.005 for 2mL; average cell-to-cell distance: 37.2 μm) on each membrane to be placed on Hypho minimal agar. For Fig. S4D, we also doubled total density to roughly 1×10^7 *E. coli* CFU/membrane.
- Monoculture in Liquid: 5×10^7 *E. coli* CFU/mL (OD_{600}=0.01 mL^{-1}; average cell-to-cell distance: 54.2 μm) in 5 mL shaken Hypho liquid minimal medium.
- Mutualism on Membranes: 5×10^6 CFU/membrane cells for both species (OD_{600}=0.005 for 2mL for *E. coli* and OD_{600}=0.0025 for 2mL for *S. enterica*; average cell-to-cell distance: 26.3 μm) on each membrane.
- Mutualism in Liquid: 5×10^7 CFU/mL for both species (OD_{600}=0.01 mL^{-1} for *E. coli* and OD_{600}=0.005 mL^{-1} *S. enterica*; average cell-to-cell distance: 43.1 μm) in 5 mL shaken Hypho liquid medium.

Monoculture in shaken liquid:

In shaken liquid, using OD_{600}=0.01/mL led to a cell density of $\rho_L = 5 \times 10^7$ CFU/mL for a cell number of $N_L = 5 \times 10^7$ in a volume of $V = 1$ cm^3. Assuming that cells were uniformly distributed, this means that each cell should be at the center of a very small square lattice with edge length, x_{LM}, and volume V_L. As a result, we estimate that individual volume of the small lattice is $V_L = \frac{V}{N_L} = \frac{1}{5 \times 10^7} = 2 \times 10^{-8}$ cm^3. Then the average distance between cells is $x_{LM} = 27.1$ μm.

Monoculture on surfaces:

The diameter of each nitrocellulose membrane was $d = 4.7$ cm, so the area for each membrane is $A_M = 17.34$ cm^2. Using a density of ~5×10^6 CFU/membrane for each species. This gives a cell density of $\rho_M = 2.88 \times 10^5$ cells/cm^2. Assuming that all cells were uniformly distributed, we estimate that each cell is at the center of a very small square with edge length, x_{MM}, and area A_M. As above, we estimate that $A_M = \frac{1}{2.88 \times 10^5} = 3.47 \times 10^{-6}$ cm^2. Then the average distance between cells is $x_{MM} = 18.6$ μm.

Mutualism in liquid:

In shaken liquid, using $\rho_L = 5 \times 10^7$ CFU/mL for both species yielded a total cell number of $N_{LC} = 1 \times 10^8$ in a volume of $V = 1$ cm^3. Assuming that cells were uniformly distributed, this means that each cell should be at the center of a very small square lattice with edge length, x_{LC}, and volume V_{LC}. As a result, we estimate that individual volume of the small lattice is $V_{LC} = \frac{V}{N_{LC}} = \frac{1}{1 \times 10^8} = 1 \times 10^{-8}$ cm^3. Then the average distance between cells is $x_{LC} = 21.5$ μm.

Mutualism on surfaces:

The diameter of each nitrocellulose membrane was $d = 4.7$ cm, so the area for each membrane is $A_M = 17.34$ cm^2. Using a density of ~5×10^6 CFU/membrane for each species, we have a total cell density of $\rho_{MC} = 5.77 \times 10^5$ cells/

cm² because we have two species. Assuming that all cells were uniformly distributed, we estimate that each cell is at the center of a very small square with edge length, x_{MC}, and area A_{MC}. As above, we estimate that $A_{MC} = \frac{1}{5.77 \times 10^5} = 1.734 \times 10^{-6}$ cm². The average distance between cells is then $x_{MC} = 13.2$ µm.

Clearly, the average cell-to-cell distances in the four conditions above were all comparable.

2. Challenges with antibiotic tolerance measurement by MDK99

In Fridman et al. (2014) and Brauner et al. (2017), tolerance was measured as the minimal duration of killing 99% of the population (MDK99) by fitting an exponential line between the two close-by time points that cover the Survival Fraction = 10^{-2} data point.

However, by definition, this metric fails to produce reliable tolerance measurements when the persister fraction is higher than 1% (Fig. S2B-C). In particular, the challenge with MDK99 is that it can still be dependent on the persister fraction, so persistence and tolerance become non-independent variables. Thus, one has to measure tolerance for the non-persister population as the "Standardized MDK99" by considering the measured persister fraction (see below). If the persister frequency is measured as p with an experimentally measured MDK99 of M, then the population tolerance is defined as:

$$\text{Tolerance} = \begin{cases} M & p \leqslant 10^{-2} \\ M \cdot \frac{f\left(10^{-4}\right)}{f(p)} & p > 10^{-2} \end{cases},$$

where $f\,(p)$ is the function in Fig. S2C that maps a persister fraction, p, to the theoretical MDK99 measurement with no change to the non-persisters' tolerance.

3. Nondimensionalization of the PDE model

We performed nondimensionalization for both sets of equations to make all variables unit- and dimension-less. First, I non-dimensionalize the PDE systems in Eqs. [S1~S2] by defining $y = \frac{x}{Q}, \tau = t_0 t$, $u = \frac{E}{W}$, $s = \frac{S}{W}$, $m = \frac{M}{W}$, $a = \frac{A}{W}$, $l = \frac{L}{W}$, $\delta_m^2 = \frac{D_M}{t_0 Q^2}$, $\delta_a^2 = \frac{D_A}{t_0 Q^2}$, $\delta_l^2 = \frac{D_L}{t_0 Q^2}$, and $k_i = \frac{K_i}{W}$ for $i \in \{M, L, A\}$. Here, I let $t_0 = t^{-1}$, $Q = 1$ unit of distance, and $W = 1$ cell unit per mL as in Hammarlund et al. (2021). Therefore, the non-dimensionalized, cross-feeding system in Eq. [S1], becomes:

$$\frac{\partial u}{\partial \tau} = \frac{r_E}{t_0}\left(\frac{m}{m + k_M}\right)\left(\frac{l}{l + k_L}\right) u - \frac{\kappa_E}{t_0} u, \quad \text{[S3a]}$$

$$\frac{\partial s}{\partial \tau} = \frac{r_S}{t_0}\left(\frac{a}{a + k_A}\right) s - \frac{\kappa_S}{t_0} s, \quad \text{[S3b]}$$

$$\frac{\partial m}{\partial \tau} = \delta_m^2 \nabla^2 m + \frac{p_M r_S}{t_0}\left(\frac{a}{a + k_A}\right) s - \frac{c_E}{t_0}\left(\frac{m}{m + k_M}\right)\left(\frac{l}{l + k_L}\right) u - \frac{\kappa_M}{t_0} m, \quad \text{[S3c]}$$

$$\frac{\partial a}{\partial \tau} = \delta_a^2 \nabla^2 a + \frac{p_A r_E}{t_0} \left(\frac{m}{m + k_m} \right) \left(\frac{l}{l + k_l} \right) u - \frac{c_S}{t_0} \left(\frac{a}{a + k_a} \right) s - \frac{\kappa_A}{t_0} a, \qquad \text{[S3d]}$$

$$\frac{\partial l}{\partial \tau} = \delta_l^2 \nabla^2 l - \frac{c_L}{t_0} \left(\frac{m}{m + k_M} \right) \left(\frac{l}{l + k_L} \right) u - \frac{\kappa_L}{t_0} l. \qquad \text{[S3e]}$$

The boundary conditions become:

$$\frac{\partial n}{\partial y} = 0 \text{ at } y = 0,\ 5, \text{ for } n \in \{u, s, m, a, l\}. \qquad \text{[S3f]}$$

Similarly, the non-dimensionalized monoculture system of E. coli in Eq. [2] becomes:

$$\frac{\partial m}{\partial \tau} = \delta_m^2 \nabla^2 m - \frac{c_E}{t_0} \left(\frac{m}{m + k_M} \right) \left(\frac{l}{l + k_L} \right) u - \frac{\kappa_M}{t_0} m, \qquad \text{[S4a]}$$

$$\frac{\partial u}{\partial \tau} = \frac{r_E}{t_0} \left(\frac{m}{m + k_M} \right) \left(\frac{l}{l + k_L} \right) u - \frac{\kappa_E}{t_0} u, \qquad \text{[S4b]}$$

$$\frac{\partial l}{\partial \tau} = \delta_l^2 \nabla^2 l - \frac{c_L}{t_0} \left(\frac{m}{m + k_M} \right) \left(\frac{l}{l + k_L} \right) u - \frac{\kappa_L}{t_0} l, \qquad \text{[S4c]}$$

with similar boundary conditions:

$$\frac{\partial n}{\partial y} = 0 \text{ at } x = 0,\ 5, \text{ for } n \in \{u, m, l\}. \qquad \text{[S4d]}$$

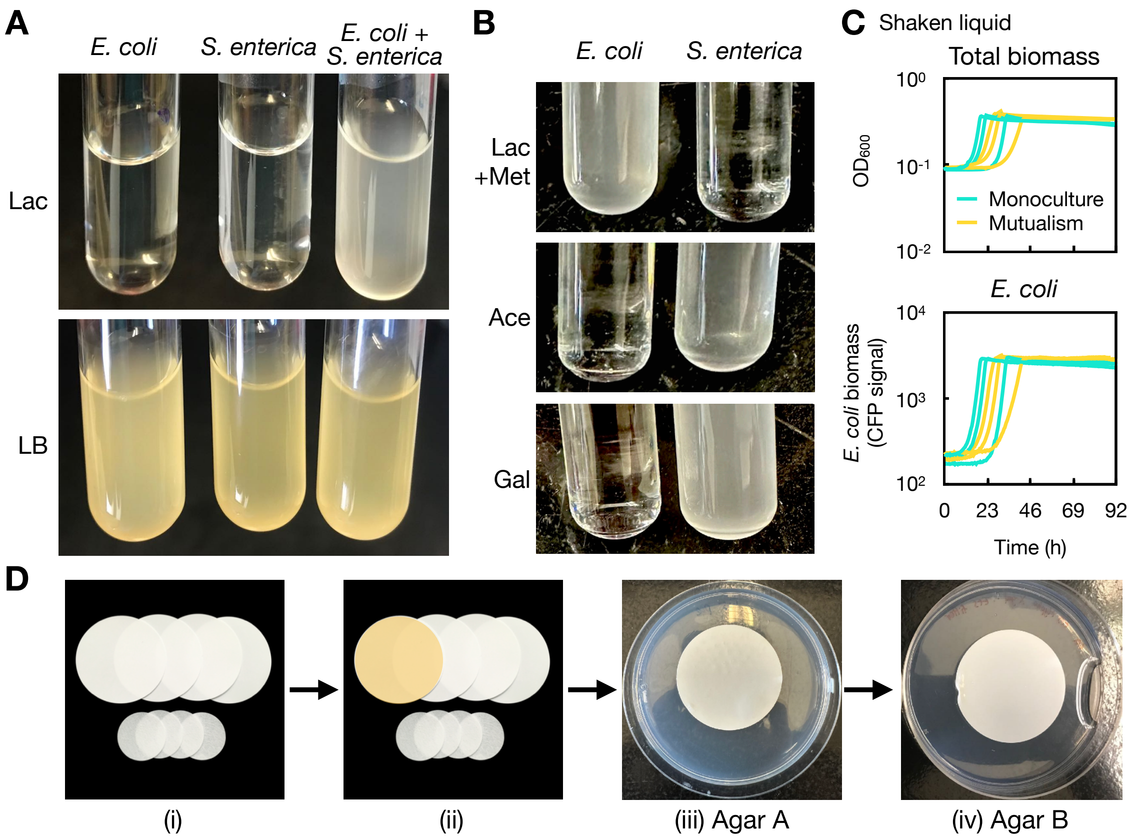

Figure S1. An obligate microbial mutualism between *Escherichia coli ΔmetB* and the methionine-overproducer *S. enterica* in the lactose minimal media. **A** Obligate mutualism is observed between the two engineered *E. coli* and *S. enterica* strains (Harcombe, 2010; Harcombe et al., 2014) in the Hypho minimal medium with lactose (Lac) as the only carbon source. Both species can be recovered in LB media. **B** The *E. coli* and *S. enterica* can also be studied in their respective monocultures while maintaining similar physiology as in the mutualistic coculture. In monoculture, *E. coli* can grow in Hypho minimal media supplemented with lactose and methionine (Met), whereas *S. enterica* can grow in Hypho minimal media supplemented with acetate (Ace) or galactose (Gal), which are at least two carbon compounds we believe *E. coli* secretes in the mutualistic coculture (Harcombe, 2010; Harcombe et al., 2018). Either species cannot grow alone in the other media due to auxotrophy. **C** When supplemented with 0.08 mM methionine and 2.78 mM lactose, *E. coli* in monoculture liquid media can grow to the same final yield as the mutualistic coculture supplemented with 2.78 mM lactose (Materials & Methods). **D** Experimental setup. On sterile nitrocellulose filter membranes (i), bacteria can be randomly distributed and immobilized by running a washed and diluted liquid culture through a membrane on a sterile funnel (ii). Then the membrane with bacteria (shade in orange) can be moved among multiple agar plates [e.g. including Agar A (iii) and Agar B (iv)]. Image of membranes in (i) and (ii) were retrieved from the manufacturer website.

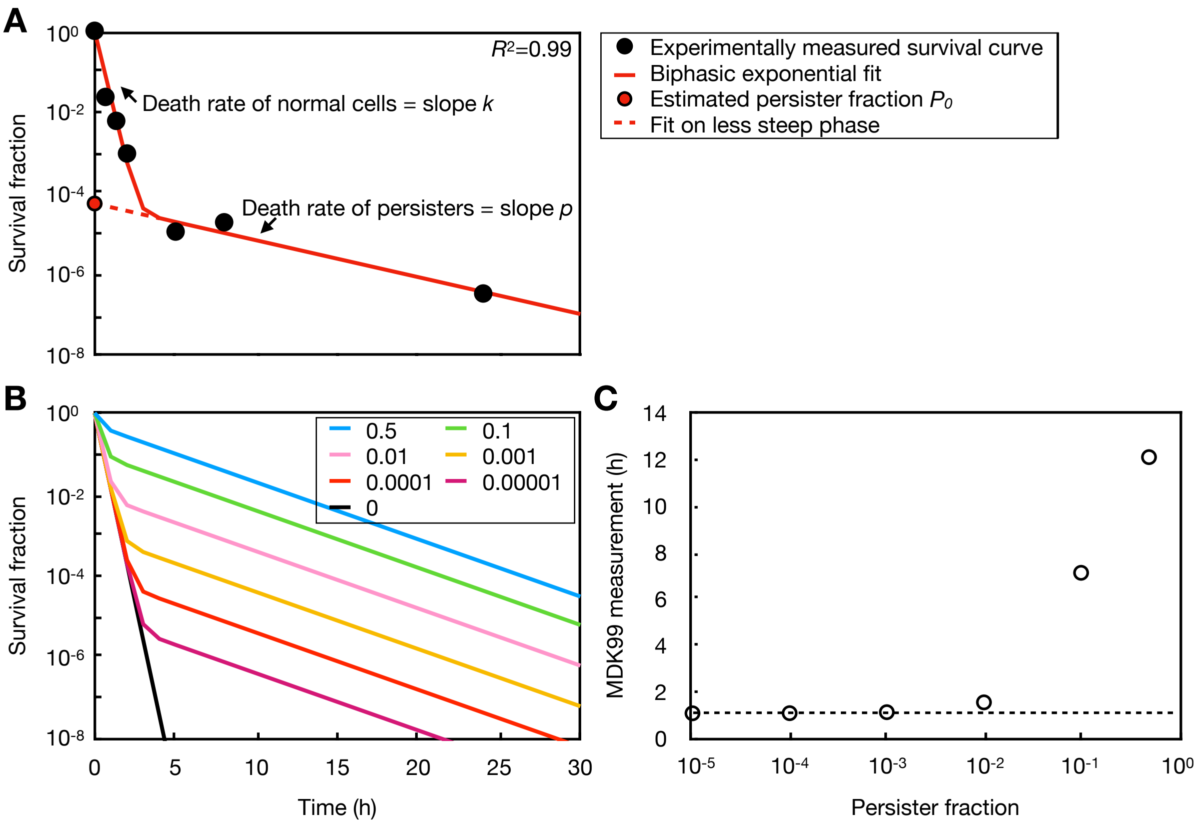

Figure S2. Measuring antibiotic tolerance and persistence. A For an experimentally measured survival curve over time (black dots), we applied a biphasic exponential line (red line) to fit the experimentally measured survival curve (one example shown), and then the intersection (red dot) between the second phase of the line (dashed line) and the *y*-axis is considered the initial persister fraction. Using this approach, tolerance is measured as inverse of the non-persister death rate (k^{-1}), whereas persistence is measured as the initial persister fraction (P_0). **B-C** Tolerance measured as the "MDK99" is inaccurate. By definition, the regular MDK99 metric becomes inaccurate in measuring non-persisters' antibiotic tolerance when persister frequency becomes higher than 1%. **B** A simple ordinary differential equation (ODE) model (Supplementary Mathematical Models 2) was run to portray antibiotic killing of bacterial populations with persisters dying 10-times more slowly than the non-persisters. The model assumes no transition between persisters and non-persisters. Survival curves were simulated with different persister fractions at t=0 (color and legend). **C** The persister fraction affects MDK99's ability to portray antibiotic tolerance. Direct MDK99 calculations were made using the simulated data as in **B**. No change was made to the non-persisters' tolerance in these simulations. Clearly, when persister fractions are above 1%, the MDK99 metric was inflated and fails to accurately measure how non-persisters respond to antibiotic killing (i.e. tolerance).

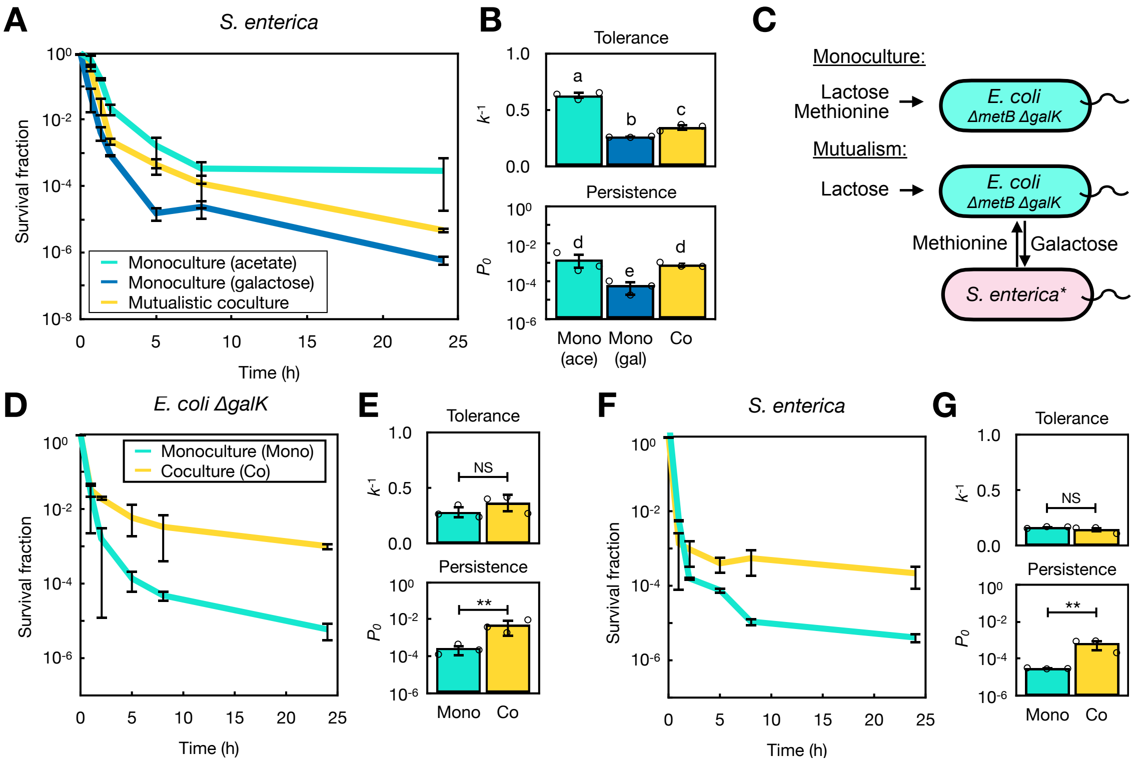

Figure S3. Antibiotic persistence also tends to be higher in *S. enterica* on the mutualistic agar. A *S. enterica* shows differential survival curves in the antibiotic treatment between the mutualistic coculture and the monoculture media with galactose (gal) or acetate (ace). **B** Using the same method as in Fig. 1C-D and S2, we found that *S. enterica* has a magnitude higher persistence in the mutualistic coculture (Mutual) than in monoculture with galactose (Mono Gal) (P=0.017), but not than the acetate monoculture (P=0.94). Although we know acetate (Harcombe, 2010) and galactose (Harcombe et al., 2018) are carbon sources that *S. enterica* consumes in the mutualistic coculture, they are not the sole source and the complete identity of carbon exchanged in this mutualism remains undefined (Martinson et al., 2023). This lack of clarity about the exact carbon source is a reason that we focused on *E. coli*. **C** A slightly different synthetic mutualism involving our *S. enterica* and an *E. coli* strain with both Δ*metB* Δ*galK* mutations. Here *E. coli* has an extra Δ*galK* mutation and no CFP gene compared with our *E. coli* strain used elsewhere in this study. We refer to this new strain "*E. coli* Δ*galK*". This *E. coli* strain cannot use the galactose it produces when degrading lactose and therefore its secretions are dominated by galactose (Harcombe et al., 2018). This *E. coli* strain still gains methionine from *S. enterica*. **D** Differential survival curves of *E. coli* Δ*galK* in monoculture (Mono) and in mutualism (Co) with *S. enterica*. **E** *E. coli* Δ*galK* has similar tolerance (P=0.26) but about an order of magnitude higher persister fraction on mutualistic agar than in monoculture (P=0.0084). **F** Differential survival curves of in *S. enterica* monoculture (Mono) and in mutualism (Co) with *E. coli* Δ*galK*. **G** *S. enterica* has similar tolerance (P=0.32) but about an order of magnitude higher persister fraction on mutualistic agar than in monoculture (P=0.0038).

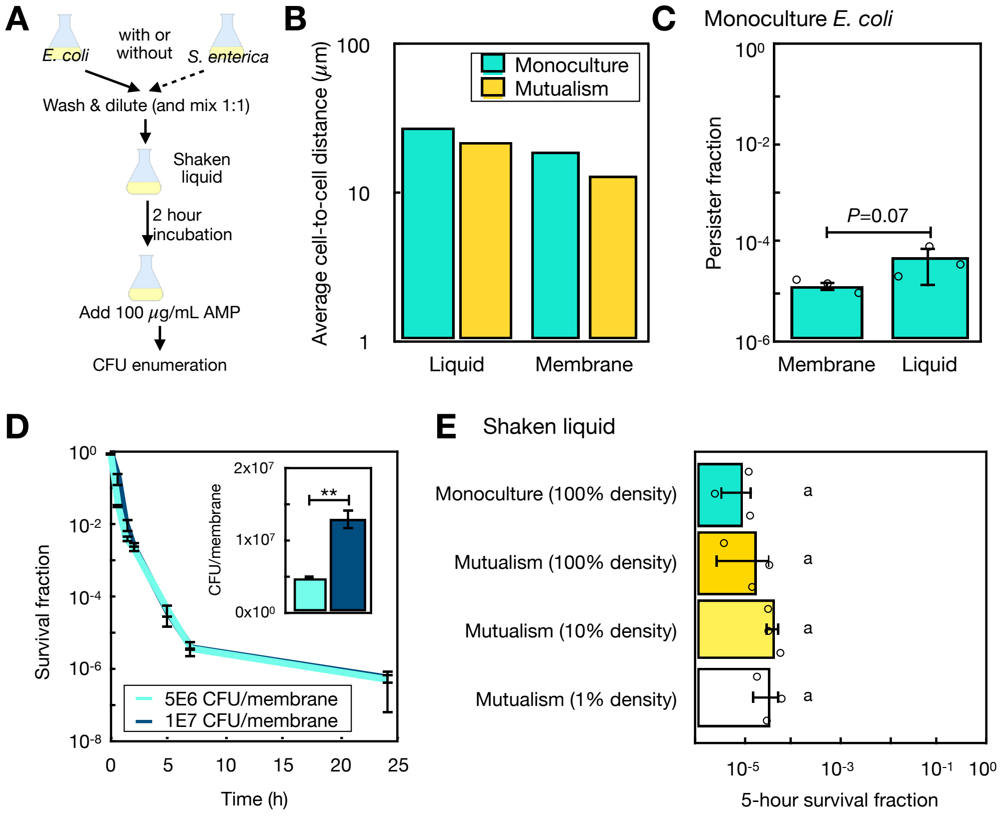

Figure S4. High persistence in the mutualistic *E. coli* on agar is not likely caused by moving from liquid to agar or average methionine starvation. A To test whether cross-feeding alone was sufficient to cause high *E. coli* persistence, we repeated the experiment in Fig. 1B in shaken liquid. **B** In all scenarios tested in this work, the average *E. coli* cell-cell distance was on the same order of magnitude. We assumed that on membranes (in liquid), each bacterial cell is at the center of a small square (cube) with identical edge length, with all of the squares (cubes) occupying the entire nitrocellulose membrane surface (liquid culture volume). The average cell-cell distance was then calculated as the distance between the center points of two adjacent squares (cubes) (Supplementary Discussion 1). **C** For log-phase monoculture *E. coli*, the difference in persister frequency between on nitrocellulose membranes and in shaken liquid is borderline significant (P=0.07). **D** Doubling *E. coli* density on monoculture agar does not affect shape of the survival curve. In the two experimental groups, the only difference is the two-fold difference between initial *E. coli* density fixated on agar (P=0.0059). **E** Reducing total methionine production in the mutualistic coculture in liquid medium to 1% original density does not affect antibiotic survival after 5-hour ampicillin killing (all pairwise Tukey's HSD P>0.19).

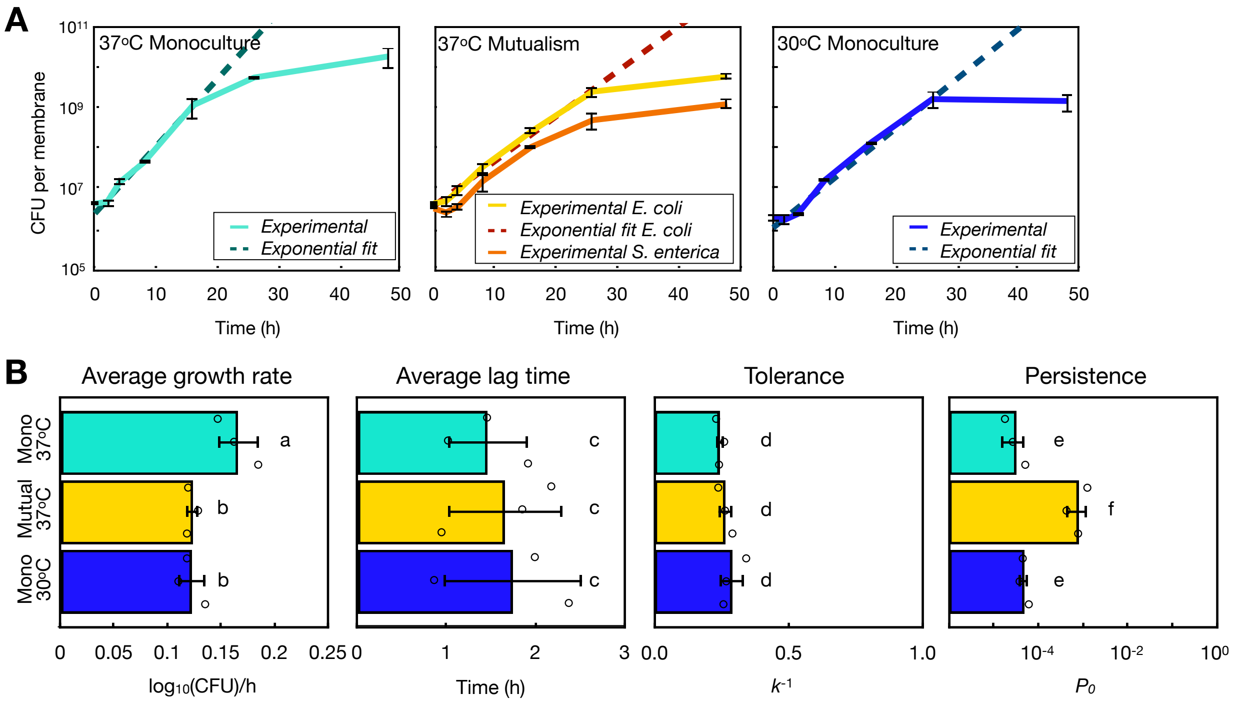

Figure S5. *E. coli* growth on nitrocellulose membranes at different temperatures. A *E. coli* growth curves on nitrocellulose membranes in various growth conditions were plotted. Cells were immobilized on nitrocellulose membranes as in Fig. 1D and S1D, and incubated statically at 37 °C or 30 °C as specified in the figure. At 2, 4, 8, 16, 26, 48 hours, membranes from each biological replicate were taken off from the agar and rinsed in 5 mL saline for CFU enumeration (disruptive sampling). Growth rate and lag time were measured by fitting a log-linear curve using custom code (Materials & Methods). **B** The average *E. coli* growth rate and lag time was assessed for populations grown on nitrocellulose membranes at 37 °C and 30 °C in monoculture (Mono) and the mutualistic coculture (Mutual). The antibiotic tolerance and persistence were also measured. On surface, *E. coli* grows faster in monoculture at 37 °C than in the mutualistic coculture (Tukey's HSD P=0.021), and than in monoculture at 30 °C (Tukey's HSD P=0.020). Across three conditions we observed, we did not find detectable difference in mean lag time in *E. coli* (pairwise Tukey's HSD P>0.85) or mean tolerance (pairwise Tukey's HSD P>0.26). High persistence was only observed in the mutualistic *E. coli* at 37 °C (pairwise Tukey's HSD P<0.00077). The *E. coli* antibiotic killing experiment on agar in Fig. 2A, 2B and S5B here were performed together.

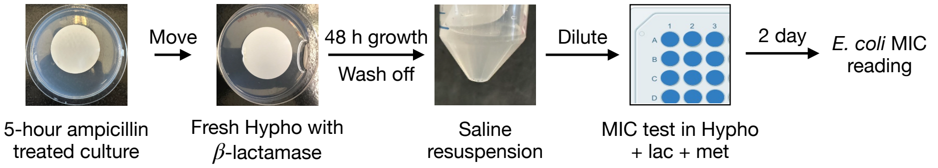

Figure S6. Schematic testing MIC of ampicillin for *E. coli* treated for 5 hours on monoculture or the mutualistic agar. For six *E. coli* and *S. enterica* biological replicates each (*n*=6), we repeated antibiotic killing on monoculture agar and the mutualistic coculture agar with *S. enterica*. After 5-hour antibiotic killing, we moved each membrane to a different agar spread with 75 μL of 125 unit/mL beta-lactamase and incubated for 2 days to obtain full growth. We washed off the membranes in saline and measured OD_{600} in saline. To test the *E. coli* MIC for these cultures, we diluted each culture to OD_{600}=0.001 per species against an ampicillin gradient (100~0.098 μg/mL) in Hypho minimal media with lactose and methionine on a single row of a 96-well plate, which was incubated with 385 rpm shaking at 37 ^{o}C for 48 hours. We read the *E. coli* MIC for each row as the lowest concentration without growth.

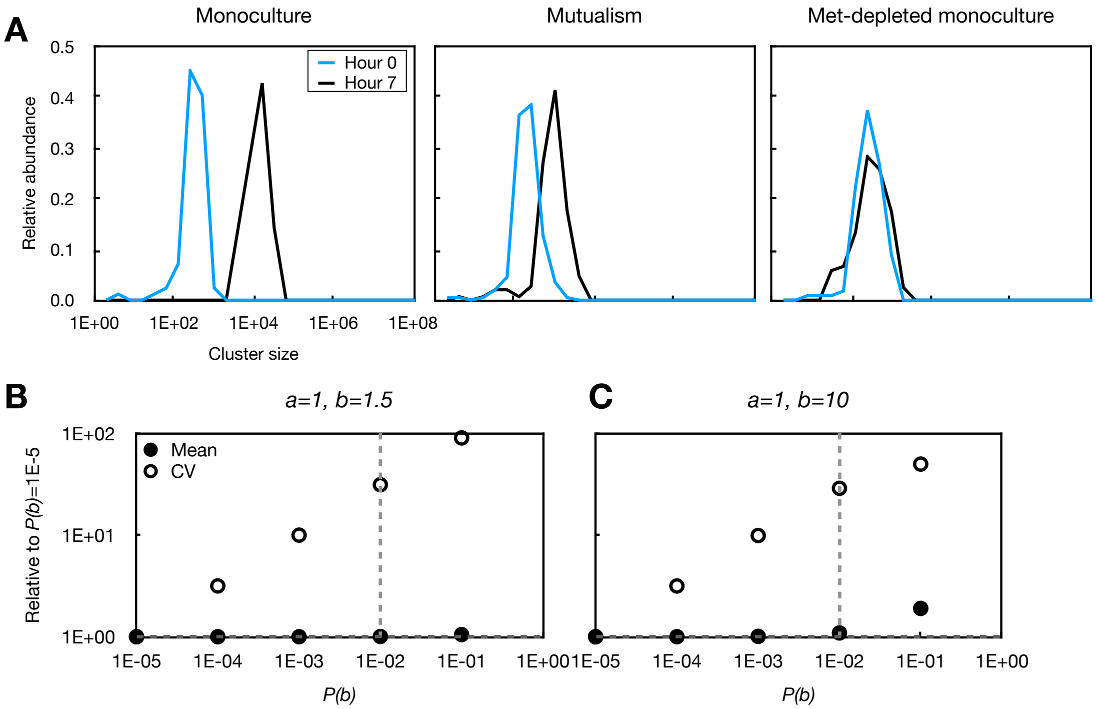

Figure S7. Tracking biomass for single *E. coli* cell clusters. A Consistent with Levin-Reisman et al. (2010), Fridman et al. (2014), Vulin et al. (2018) and Chacón et al. (2018), we measured lag time under the microscope as the time it takes for a single CFP-labeled *E. coli* cluster identified at Hour 0 to gain biomass greater than a certain threshold (in our case, to gain 10% of biomass). Consistent with our intuition, we found that by Hour 7, the *E. coli* clusters on average gain biomass by 10~100 fold in the mutualistic coculture with *S. enterica* or the monoculture, but almost no biomass was gained in the methionine- (Met-) depleted monoculture. We used the microscopic measurements in this study for single cells rather than tracking colony-level growth on agar using existing methods like ScanLag (Fridman et al., 2014), for reasons detailed in Fig. S12. **B** In Fig. 3B, we did not observe any significant difference in the mean lag time in *E. coli* between monoculture and the mutualistic coculture (P=0.36). To rationalize this lack of difference in mean but presence of difference in CV (coefficient of variation) of lag time, we built a simple mathematical model. For a dataset with 100,000 data entries all equal to a=1, we gradually changed a fraction [*P(b)*] of the entries to an outlier value b=1.5, and calculated the population mean and CV. We then plotted the relative mean and CV of each population against those at *P(b)*=1E-5. As we increased *P(b)*, we immediately saw exponential visible increase in relative CV but not in relative mean, suggesting that CV of a population is more susceptible to outliers than the mean is. Grey dashed line indicates *P(b)*=1E-2, which is roughly the mutualistic *E. coli* cell faction with longer lag time than the rest of the population we observed in Fig. 3B. **C** Having an extreme outlier for the simulation (b=10) in **B** does not change the fact that CV is more susceptible from impact from outliers than the mean is.

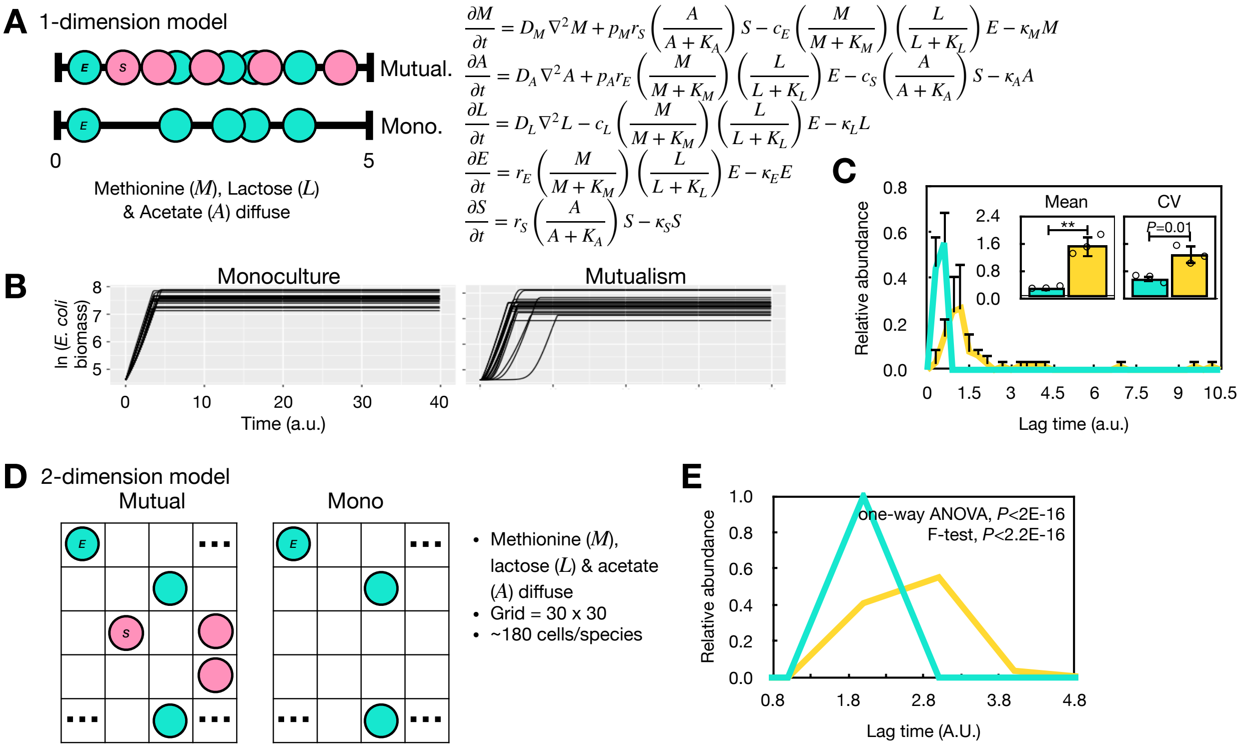

Figure S8. A simple mathematical model supports the microscopy experiment results. **A** A 1-dimension biophysical (resource-consumption, reaction-diffusion) PDE model was designed to describe *E. coli* (cyan) growth at a single colony level on a 1-dimension world with and without *S. enterica* (pink). Briefly, lactose is initially set up in the entire 1-dimension line. The *E. coli* (*E*) and *S. enterica* (*S*) cells were seeded at 20 random locations, and the cells can grow (increase biomass) by consuming nutrient. *E. coli* consumes lactose (*L*) and methionine (*M*) to grow, whereas *S. enterica* consumes acetate (*A*). *E. coli* secretes acetate as it grows, and *S. enterica* secretes methionine. The nutrient diffuses in the world at diffusion constants D_i for $i = M, A, L$ that we chose based on previous work (Bassi et al. 1987; Ma et al., 2005; Hazarika et al., 2006; Chikode et al., 2007). There is also a natural decay rate for all terms, κ_i for $i = M, A, L, E, S$. See Supplementary Mathematical Model 1 and Supplementary Table 1 for details. **B** When seeded at the same location in monoculture or in mutualism with *S. enterica*, the individual *E. coli* growth curves were very different. Results shown here are from one representative independent simulation from **A**. **C** Characterizing all growth curves in each simulation following Materials & Methods, we found significantly larger mean (*P*=2E-3) in the *E. coli* lag time in the cross-feeding coculture than in monoculture, and this was mainly caused by a wider and right-shifted lag time distribution (coefficient of variation, or CV, was higher, *P*=0.01). **D** The PDE model was also implemented in a 2-dimension world using the finite difference method. Otherwise the parameters and implementation are consistent with that in **A**. **E** In a 2-dimension world, *E. coli* in the mutualistic coculture has higher variance in the lag times than in monoculture (F-test, *P*<2.2E-16).

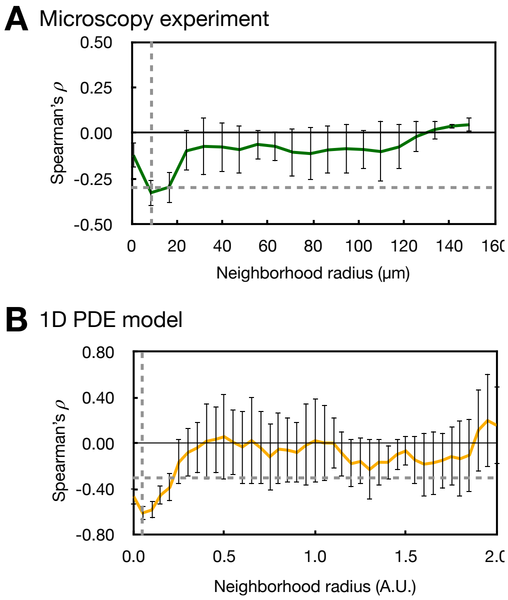

Figure S9. *E. coli* and *S. enterica* typically interact in small neighborhoods. Here we used a modified method from Dal Co et al. (2020) to measure the interaction range between *E. coli* and *S. enterica* in the mutualistic coculture. In brief, we calculated the initial *S. enterica* biomass fraction in neighborhoods with various sizes (radius), and correlated this biomass fraction at all radius sizes with the *E. coli* cell cluster lag time (Fig. 3B-C). Then, the neighborhood radius was plotted against the Spearman's ρ in **A-B**. The horizontal gray dashed line denotes Spearman's ρ=-0.3. The vertical gray dashed line indicates the radius size where the correlation was best, which was defined as the interaction range (Dal Co et al., 2020). Error bars denote standard deviation of the average measurements from 3 biologically independent trials. **A-B** Both the microscopy experiment and the 1-dimension PDE model revealed that *E. coli* and *S. enterica* usually interact in very small neighborhoods (~10 μm in experiment, Fig. 3C). However, the correlation in the 1-dimension PDE model (Spearman's ρ=-0.610±0.0633) is much better than in the microscopy experiment (ρ=-0.327±0.0735, P<0.002, n=3).

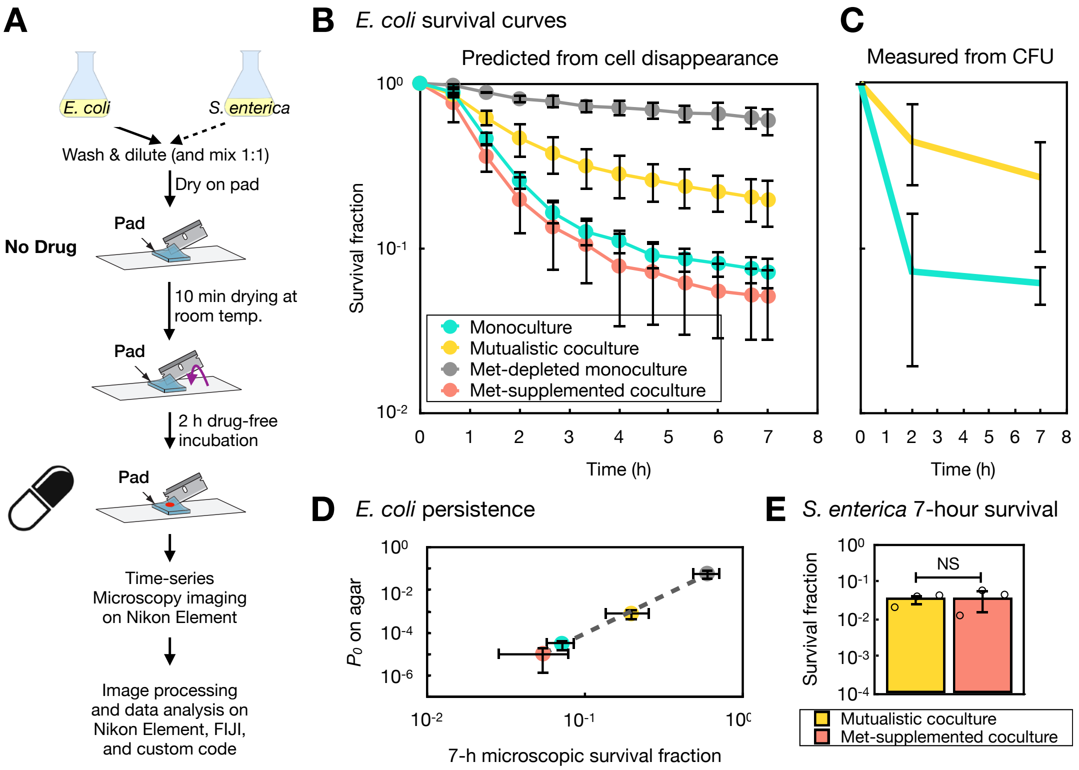

Figure S10. Survival curves of *E. coli* measured by single-cell level death time are consistent with that by CFU counts over time. A A fluorescent microscopy setup to track the *E. coli* ampicillin-induced death with spatial structure. In this panel, all illustrations that contain agarose pads were edited from the original one in Skinner et al. (2013). A 1.5 µL cell droplet of OD_{600}=0.05 from saline-washed *E. coli* and OD_{600}=0.025 of *S. enterica* was first incubated on top of the Hypho minimal agarose pad to dry at room temperature, and then flipped over (purple arrow) on a microscopic slide for incubation at 37 ^{o}C. We have tested that this concentration leads to similar cell density in the microscopic frame as on the nitrocellulose membrane setup. Afterwards, a 1.5 µL ampicillin droplet was added on top of the pad (red dot) such that the entire agarose pad will eventually reach 100 µg/mL. **B** Repeating the experiment in four conditions in Fig. 1H and 2B on the microscope recreated the survival curves for *E. coli* that shared the same trends. **C** Survival curves plotted for monoculture and the mutualistic coculture *E. coli* by CFU counting over time matched that measured by single-cell death time. **D** The mean survival fraction data after a 7-hour ampicillin treatment under the agarose pad can predict the mean persister fraction measurements on nitrocellulose membranes in Fig. 1F and Fig. S6 (Adjusted R^2=0.91, P=0.030). **E** The mutualistic (yellow) and the methionine- (Met-) supplemented (red) cocultures led to similar *S. enterica* responses to the ampicillin treatment. We found that the differences in the 7-hour survival fractions in *S. enterica* between the two conditions were borderline different (P=0.95).

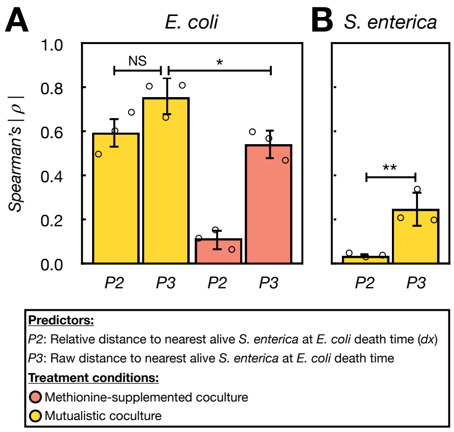

Figure S11. Removing (*P3*) the standardization process on the predictor *P2* increases predictability for single-cell death time. In using predictor *P2*, we considered the relative distance of a focal cell to its nearest alive partner to predict the focal cell's death time under ampicillin killing, where the relative distance is done by standardizing raw cell distance against the average distance measured given the total number of remaining cells in the view at each time point. Although our goal in using *P2* rather than *P3* was to prevent autocorrelation as fewer cells will be present in the frame as more cells die, we may also artificially decrease the distance between partners at later time points and increase the distance between partners at earlier timepoints. Here we showed predictability of *P3*. **A** For *E. coli*, *P3* is a better predictor than *P2* in the methionine-supplemented coculture condition (Tukey's HSD *P*=0.00057), but *P3*'s superiority in the mutualistic *E. coli* was not detected (Tukey's HSD *P*=0.11). Removing this standardization does not change our conclusion, because *P3* is still a better predictor for the methionine-supplemented than the mutualistic *E. coli* (Tukey's HSD *P*=0.035). **B** For the mutualistic *S. enterica*, removing the standardization procedure and using predictor *P3* also led to better, and more significant predictability (*P*=0.0099).

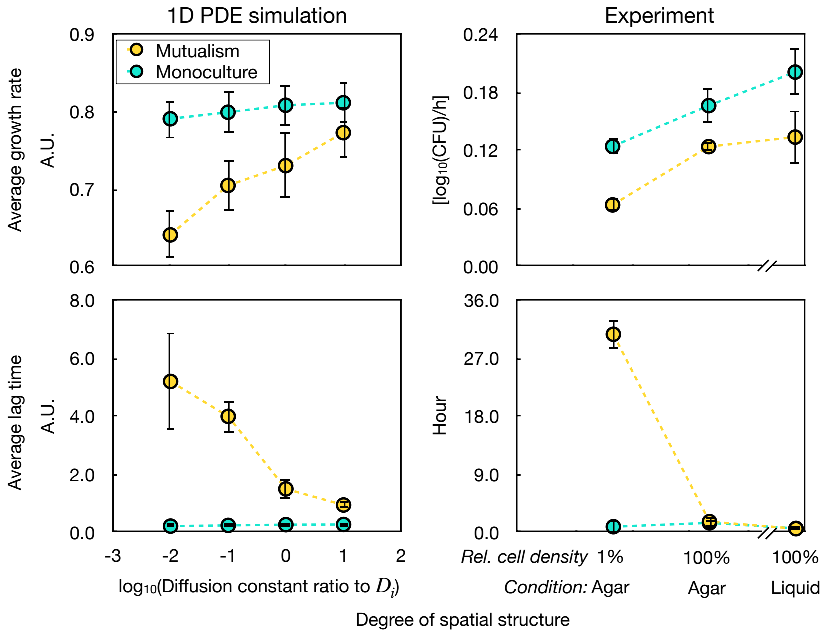

Figure S12. Choosing microscopy over the ScanLag approach to study sub-population growth of *E. coli*. ScanLag is a method where the growth of bacteria is tracked on a single-colony level on agar plates on an office scanner that periodically takes images of the plate (Levin-Reisman et al., 2010). This established method has been successfully implemented in numerous studies (e.g. Fridman et al., 2014; Levin-Reisman et al., 2017; Chacón et al., 2018; Vulin et al., 2018) to understand individual heterogeneity of a given bacterial population. We decided to perform fluorescent microscopy rather than this method because this method was shown (Levin-Reisman et al., 2010; Fridman et al., 2014) to work best with 100~200 colonies on an agar plate with a diameter of 10 cm. This density is about 10^{-5} times less than on the nitrocellulose membrane setting in the current work. Both the 1-dimension PDE model and our experiments show that lowering cell density will artificially augment the mean of the lag time for *E. coli*.

Parameter	Unit	Value	Biological interpretation	Source
x	Arbitrary unit of distance	1.00	Unit of space.	Definition
t	Arbitrary unit of time	1.00	Unit of time.	Definition
Y	Arbitrary unit of distance	5.00	Total size of the 1-dimension modeling canvas.	Definition
E, S, M, A, L	Cell unit/mL	-	*E. coli*, *S. enterica*, methionine, acetate, and lactose, respectively.	Definition; Hammarlund et al. (2021)
D_M	x^2/t	0.01	Diffusion constant of methionine.	Estimated to be small than D_L based on Ma et al. (2005) and Hazarika et al. (2006)
D_A, D_L	x^2/t	0.05	Diffusion constants of the acetate and lactose sugars.	Estimated based on Bassi et al. (1987) and Chikode et al. (2007)
p_M	Unitless	1.56	Production rate of methionine by *S. enterica*.	Estimated based on Hammarlund et al. (2021)
p_A	Unitless	1.01	Production rate of acetate by *E. coli*.	Hammarlund et al. (2021); adjusted with unpublished laboratory data
c_E	t^{-1}	0.10	*E. coli* consumption rate of methionine.	Estimated based on Hammarlund et al. (2021)
c_L	t^{-1}	1.00	*E. coli* consumption rate of lactose.	Hammarlund et al. (2021)
c_S	t^{-1}	1.00	*S. enterica* consumption rate of acetate.	Hammarlund et al. (2021)
r_E	t^{-1}	1.00	*E. coli* maximum growth rate.	Hammarlund et al. (2021)
r_S	t^{-1}	0.50	*S. enterica* maximum growth rate.	Hammarlund et al. (2021)
K_M, K_A, K_L	Cell unit/mL	1.00	Half-saturation methionine, acetate, lactose concentration for bacterial growth.	Hammarlund et al. (2021)
κ_E, κ_S, κ_M, κ_A, κ_L	t^{-1}	5.00E-09	Natural decay rate of *E. coli*, *S. enterica*, methionine, acetate, and lactose.	Estimated and adjusted for simplicity

Supplementary Table 1. Parameters used in the PDE models.

Supplementary References

Bassi AS, Rohani S, Macdonald DG. (1987). Measurement of effective diffusivities of lactose and lactic acid in 3% agarose gel membrane. *Biotechnology & Bioengineering* **30**(6), 794-7.

Brauner A, et al. (2017). An Experimental Framework for Quantifying Bacterial Tolerance. *Biophysics Journal* **112**, 2664-2671.

Chacón JM, et al. (2018). The spatial and metabolic basis of colony size variation. *ISME Journal* **12**, 669-680.

Chikode PP, Pawar SJ, Fulari VJ, Dongre MB. (2007). Determination of Diffusion Coefficient of Lactose Solution. *Journal of Holography and Speckle* **4**(1):19-25.

Dal Co A, et al. (2020). Short-range interactions govern the dynamics and functions of microbial communities. *Nature Ecology & Evolution*, **4**(3), 366-375.

Fridman O, et al. (2014). Optimization of lag time underlies antibiotic tolerance in evolved bacterial populations. *Nature* **513**, 418-421.

Hammarlund SP, et al. (2019). A shared limiting resource leads to competitive exclusion in a cross−feeding system. *Environmental Microbiology* **21**, 759-771.

Hammarlund SP, et al. (2021). Limitation by a shared mutualist promotes coexistence of multiple competing partners. *Nature Communications* **12**, 1-8.

Harcombe WR. (2010). Novel cooperation experimentally evolved between species. *Evolution* **64**, 2166-2172.

Harcombe WR, et al. (2014). Metabolic resource allocation in individual microbes determines ecosystem interactions and spatial dynamics. *Cell Reports* **7**, 1104-1115.

Harcombe WR, et al. (2018). Evolution of bidirectional costly mutualism from byproduct consumption. *Proceedings of the National Academy of Science of the United States of America*. **115**, 12000-4.

Levin-Reisman I, et al. (2010). Automated imaging with ScanLag reveals previously undetectable bacterial growth phenotypes. *Nature Methods* **7**, 737-39.

Levin-Reisman I, et al. (2017). Antibiotic tolerance facilitates the evolution of resistance. *Science* **355**, 826-30.

Hazarika S, et al. (2006). A Quantitative Structure Activity Relationship Study on Permeation of Amino Acids in Enantioselective Membranes. *Journal of Applied Membrane Science & Technology* **2**, 13-29.

Ma Y, et al. (2005). Studies on the Diffusion Coefficients of Amino Acids in Aqueous Solutions. *Journal of Chemical & Engineering Data* **50(4)**, 1192–1196.

Martinson JNV, et al. (2023). Mutualism reduces the severity of gene disruptions in predictable ways across microbial communities. *ISME Journal* **17**, 2270–2278.

R Core Team. (2024). R: A language and environment for statistical computing. R Foundation for Statistical Computing, Austria.

Skinner SO, et al. (2013). Measuring mRNA copy number in individual *Escherichia coli* cells using single-molecule fluorescent *in situ* hybridization. *Nature Protocol* **8**(6), 1100-1113.

Sun Z, et al. (2019). Microbial cross-feeding promotes multiple stable states and species coexistence, but also susceptibility to cheaters. *Journal of Theoretical Biology* **465**, 63-77.

Vulin C, et al. (2018). Prolonged bacterial lag time results in small colony variants that represent a sub-population of persisters. *Nature Communications* **9**: 4074.
